# The Utilisation of Solid Fuels Derived from Waste Pistachio Shells in Direct Carbon Solid Oxide Fuel Cells

**DOI:** 10.3390/ma14226755

**Published:** 2021-11-09

**Authors:** Magdalena Dudek, Bartosz Adamczyk, Przemysław Grzywacz, Radosław Lach, Maciej Sitarz, Magdalena Leśniak, Marcin Gajek, Krzysztof Mech, Małgorzata Wilk, Alicja Rapacz-Kmita, Magdalena Ziąbka, Piotr Dudek

**Affiliations:** 1 Faculty of Energy and Fuels, AGH University of Science and Technology, Av. Mickiewicza 30, 30-059 Kraków, Poland; bartosz.adamczyk@agh.edu.pl (B.A.); grzywacz@agh.edu.pl (P.G.); 2 Faculty of Materials Science and Ceramics, AGH University of Science and Technology, Av. Mickiewicza 30, 30-059 Kraków, Poland; radoslaw.lach@agh.edu.pl (R.L.); msitarz@agh.edu.pl (M.S.); mlesniak@agh.edu.pl (M.L.); mgajek@agh.edu.pl (M.G.); kmita@agh.edu.pl (A.R.-K.); ziabka@agh.edu.pl (M.Z.); 3 Academic Centre for Materials and Nanotechnology, AGH University of Science and Technology, al. A. Mickiewicza 30, 30-059 Kraków, Poland; kmech@agh.edu.pl; 4 Faculty of Metals Engineering and Industrial Computer Science, AGH University of Science and Technology, Av. Mickiewicza 30, 30-059 Kraków, Poland; mwilk@agh.edu.pl; 5 Faculty of Mechanical Engineering and Robotics, AGH University of Science and Technology, Av. Mickiewicza 30, 30-059 Kraków, Poland; pdudek@agh.edu.pl

**Keywords:** direct carbon solid oxide fuel cells, pistachio shells, gasification, Boudouard reaction

## Abstract

The comprehensive results regarding the physicochemical properties of carbonaceous materials that are obtained from pistachio shells support their usage as solid fuels to supply direct carbon solid oxide fuel cells (DC-SOFCs). The influence of preparation conditions on variations in the chemical composition, morphology of the biochar powders, and degree of graphitization of carbonaceous materials were investigated. Based on structural investigations (X-ray diffraction analysis and Raman spectroscopy), it was observed that disordered carbon particles developed during the application of thermal treatments. The use of X-ray fluorescence enabled a comparative analysis of the chemical composition of the inorganic matter in biocarbon-based samples. Additionally, the gasification of carbonaceous-based samples vs. time at a temperature of 850 °C was investigated in a H_2_O or CO_2_ gas atmosphere. The analysis demonstrated the conversion rate of biochar obtained from pistachio shells to H_2_, CH_4_ and CO during steam gasification. The electrochemical investigations of the DC-SOFCs that were supplied with biochars obtained from pistachio shells were characterized by satisfactory values for the current and power densities at a temperature range of 700–850 °C. However, a higher power output of the DC-SOFCs was observed when CO_2_ was introduced to the anode chamber. Therefore, the impact of the Boudouard reaction on the performance of DC-SOFCs was confirmed. The chars that were prepared from pistachio shells were adequate for solid fuels for utilization in DC–SOFCs.

## 1. Introduction

Recently, special attention was paid to direct carbon solid oxide fuel cell (DC-SOFC) technology. DC-SOFCs are electrochemical devices that directly convert the chemical energy of carbonaceous fuels into electricity and heat. In the classical conception of DC-SOFCs, carbon can be directly electrochemically oxidized to carbon dioxide via reaction (1) as follows:

C + 2O^2−^→CO_2_ + 4e^−^ (1)

Additionally, it can be formed in a sequence of the following electrochemical reactions (2) and (3):

C + O^2−^→CO + 2e^−^ (2)

CO + O^2−^→CO_2_ + 2e^−^ (3)

C + CO_2_→2CO (4)

The Boudouard reaction (4) with CO_2_ and C as reactants—can serve as an additional source of the CO that is consumed in an electrochemical reaction (3) [1,2,3]. DC-SOFCs can be supplied with a wide range of carbon-rich fuels, such as coal, coke, graphite, municipal solid waste (MSW), activated carbon, and biomass-based fuels. However, solid fuels, which are characterized by low reactivity in the Boudouard reaction, will not contribute significantly to the performance of DC-SOFCs via gasification reactions. In this situation, the performance is limited by the direct oxidation of carbon particles on the anode surface of the DC-SOFCs [4,5,6]. The effective coupling of electrochemical oxidation and the reverse Boudouard reaction maintains the higher power output P_max_ and stable operation of the DC-SOFCs, which was confirmed by theoretical simulations and experimental research [7,8,9,10,11]. Moreover, the development of DC-SOFCs creates new possibilities for the electrochemical conversion of various carbon-rich waste fuels to generate electricity and heat. One of the most attractive groups of such fuels is renewable biomass-based solid waste fuels. Biomass-based solid fuels can be divided into two groups based on their main sources, the first of which is called woody biomass-based fuels. The second group comprises the fuels that are obtained from organic biomass waste materials and by-products of industrial agricultural processes, which are suitable for conversion into DC-SOFCs [12,13].

Jang et al. demonstrated the possibility of directly converting waste coffee grounds into electricity. The DC-SOFCs exhibited a maximum power density that was twice as high as the application of carbon black as a solid fuel. The advantage of using carbon-rich organic waste as a solid fuel to supply DC-SOFCs is the presence of natural minerals in their structure, which can act as a catalyst in the Boudouard reaction. Cai et al. noticed that the suitability of orchid leaf char as a solid fuel for utilization in DC-SOFCs as the biologically accumulated Ca in leaf charcoal acts as a catalyst in such a reaction. Yu et al. found that DC-SOFCs that were supplied by the biochar that was derived from a pepper straw achieved a maximum power density of 217 mW cm^−2^. This is comparable to 252 mW/cm^2^, which is the power density of the hydrogen-fueled device at a temperature of 850 °C. Therefore, the results obtained by Yu et al. demonstrate that the pepper-straw charcoal contains natural catalysts, which contribute to improving the electrochemical oxidation of solid fuels in DC-SOFCs [14,15,16,17].

Qiu et al. investigated the feasibility of using carbon-rich biochar derived from wheat straw, corncobs, and bagasse as fuels for DC-SOFCs. The peak power densities of 187 mW/cm^2^ and 204 mW/cm^2^ at 800 °C were achieved by the fuel cells with wheat-straw charcoal and corncob charcoal, respectively, while the cell with bagasse charcoal had the highest output power of 260 mW/cm^2^ [18]. Therefore, biochars with natural catalysts are attractive, low-cost solid fuels for DC-SOFCs. The biomass-derived solid fuels have a high content of volatiles and contain a small number of substances, mainly alkali oxides and iron, as the so-called natural catalysts for the Boudouard reaction, which supports the process of electrochemical oxidation of the carbon particles [19,20,21]. The key factors that determine the operating parameters of DC-SOFCs that are powered by solid waste biomass are the physicochemical properties of biocarbon that is used as a solid fuel. In DC-SOFCs, the waste raw material can undergo various thermochemical conversion processes to produce a solid fuel, and the gases that are present during the process have a significant impact on ohmic resistance and the life of the components of solid oxide fuel cells (SOFCs) [22,23,24,25]. Previous studies successfully used walnut shells as a solid fuel to power SOFCs [26,27]. Additionally, pistachio nut shells, which are another typical waste material from the food industry, are considered refuse-derived fuels and can be used in the decentralized energy sector [28,29].

The aim of this paper is to investigate the possibility of using pistachio shells as solid fuels to supply DC-SOFCs. The focus is on characterizing the physicochemical properties of the pistachio shells, which are crucial for their use as solid fuels

## 2. Materials and Methods

### 2.1. Preparation of Waste-Biomass Solid Fuels

The pistachio shells were selected to optimize their preparation and thermal treatment to obtain the required physicochemical properties of the samples, which is crucial for their application as solid fuels in DC-SOFCs. Before being used in laboratory practice, they were washed with deionized water to remove any traces of salt, which is often used as an additive for snacks. The shells were crushed and then ground in a grinder. Small portions of the raw, ground pistachio shells were processed for further analytical and electrochemical studies as reference samples for torrefied biomass or charred samples. The torrefied biomass or chars were obtained by thermally treating the powdered shells in a quartz reactor at 200 °C to 850 °C for one hour in a nitrogen gas atmosphere. To obtain the same conditions, the temperature was ramped up at a rate of 5 °C/min until the temperature reached 850 °C. After the thermal treatment, the samples were cooled to room temperature, removed from the reactor, and ground in a mortar. The powdered solid carbon fuels were passed through a mesh, steel 0.02 mm sieve (Conbest, Kraków Poland). The carbon powders that were prepared in this way were the subject of further investigation. The following abbreviations were used throughout the text for the biomass fuel samples that were tested: the entire sample series is represented by a capital P; the numbers 200, 300, 400, 600, and 850 represent the temperatures of the thermal treatment of pistachio shell samples; and the mark P0 refers to the ground sample that has not undergone any thermal treatment.

### 2.2. Analytical Methods Used to Study the Physicochemical Properties of Solid Fuels

The raw ground pistachio shells and biochars were subjected to elemental and technical analyses. A proximate analysis (carbon, hydrogen, and sulphur) was performed using an ELTRA CHS-580 analyzer, Eltra Gmbh, Haan; Germany) The oxygen content is determined as the difference between 100% and the sum of the other determined components. The technical analysis included determining the moisture and ash and was performed using the gravimetric method. These tests were carried out in the muffle furnace (Czylok, Jastrzebie, Poland) according to the following standards: PN-EN ISO 18134-1:2015-11 and PN-EN ISO 18134-3:2015-11. The X-ray diffraction method (XRD) was used to evaluate the phase composition of raw pistachio shells and torrefied and charred carbonaceous materials. Additionally, an analysis of the inorganic material (ash) after combustion was performed to obtain helpful information regarding the phase composition of chemical compounds, which can contain biochar as inorganic matters. The XRD patterns that were recorded for carbonaceous-based materials enabled the identification of the degree of graphitization of the obtained biocarbon samples. The XRD measurements were performed using the Panalytical X’Pert Pro system with monochromatic CuK radiation. The Raman spectra of carbon particles were recorded using an FTS 6000 Bio-Rad Spectrometer with Raman spectroscopy (Nd: YAG Spectra Physics T10, 1064 nm laser), and the spectra were recorded after 10,000 scans with a resolution of 4 cm^−1^. The midinfrared spectroscopy (MIR) studies for inorganic ashes were performed using a Bruker Vertex 70v spectrometer (WITec Wissenschaftliche Instrumente und Technologie GmbH; Ulm, Germany) The standard KBr palette method was used, and 128 scans were accumulated with a resolution of 4 cm^−1^ in the range 4000–400 cm^−1^ to decipher the inorganic minerals in the ash samples. Scanning electron microscopy (Nano Nova SEM 200 FEI, Eindhoven, The Netherlands), coupled with the EDX system (EDAX, Eindhoven, The Netherlands), was used to determine the possible variations in the morphology and the presence of inorganic elements of mineral matters of the carbon samples. The chemical analysis of the inorganic elements in biochars was determined using the Wavelength Dispersive X-ray Fluorescence Spectroscopy (WDXRF) and carried out using the WDXRF Axios mAX spectrometer (PANalytical, Malvern, UK). The system uses a 4 kW rhodium tube that is equipped with a window of 30-micron thickness with a maximum accelerating voltage of 60 kV and a maximum current of 150 mA. The qualitative spectrum analysis was performed by identifying the spectral lines, and the quantitative analysis was performed using the fundamental parameters method in the range of fluorine–uranium (Na–U). The contents of the determined elements were normalized to 100% by mass.

### 2.3. Thermal Behaviour of Pistachio Shells Investigated Using a Thermal Analysis

The thermal effects that occurred during the heating of solid carbon fuel in the temperature range of 25–1000 °C in the nitrogen gas stream were measured using the Differential thermal analysis (DTA) and thermogravimetric (TG) methods (Simultaneous Thermal Analyzer—STA 449 F3Jupiter^®^). The samples (approx. 50 mg) were ramped up at a rate of 10 °C/min in a platinum crucible. The measurements were carried out in an N_2_ gas atmosphere.

The TG method was used to determine the chemical reactivity of the obtained charcoal samples with CO_2_ in the temperature range of 20–850 °C. A thermobalance device (Rubotherm DynTHERM 1100-40 MP-G analyzer, TA Instruments, Bochum, Germany) was used for investigations in the CO_2_ gas atmosphere. TG curves were recorded in a temperature range of 25–850 °C in a pure CO_2_ gas atmosphere at a pressure of 0.1 MPa (abs). The CO_2_ gas flow was 100 mL min^−1^, and the temperature ramp was 10 °C min^−1^. The final heating temperature of 850 °C was maintained for 20 min. The gasification of biochar that was obtained from pistachio shells in a H_2_O gas atmosphere was studied using the thermovolumetric method. All measurements were performed at a temperature of 850 °C. The application of the volumetric method enabled the determination of the efficiency of the conversion solid biochar sample P850 by steam, used as gasification agent for the following gaseous products: H_2_, CO, CH_4_, and CO_2_. In the same experimental conditions, the gasification process of charcoal (Merck, Germany, marked as CH-M) and Carbon Black-N221 (marked CB-221 Konimpex, Poland) using H_2_O as the gasification agent was conducted. The carbonaceous materials are often used to supply DC-SOFCs. The measurements were carried out using unique laboratory equipment that enables the kinetics of gasification under isothermal conditions to be investigated in a wide pressure range of H_2_O. This method was previously used to study the gasification of coal samples. The description of the apparatus was described in papers [30,31,32].

### 2.4. Analysis of the Chemical Stability of Anode Materials in Direct Contact with Samples of Solid Fuels P0–P850 Obtained from Pistachio Shells

The chemical reactivity of two-phase cermets: Ni-GDC and Ni-YSZ—anodes with prepared carbon-based solid fuels was investigated. Several common samples were prepared to study the chemical reactivity. The metallic nickel (Ni), cubic yttria-stabilized zirconia (YSZ) or gadolina-doped ceria (GDC) powders were mixed with powdered raw ground pistachio shells (P0), torrefied pistachio shells (P200, P300 and P400) and charred pistachio shells (P600 and P850) in a volume ratio of 1:1. The investigated series of samples from P200 to P850 and the components of the anode materials, such as Ni, YSZ and GDC, were first mixed in an agate mortar in the ethyl alcohol environment. The homogenized samples with a mass of approximately 5 g were placed in a flat Al_2_O_3_ crucible, and the samples were placed in a quartz reactor. The flat crucible was placed centrally in the quartz reactor, and the temperature was measured with a K-type thermocouple. The crucible with the tested sample remained constantly in the nitrogen stream. The ramp of temperature increase in the tube furnace was fixed (5 °C/min) from 100 °C to 850 °C. The samples (solid carbon-based fuels with YSZ, GDC or Ni additions) were then heated at 850 °C for 100 h. After that, the samples were cooled down to room temperature, and the cooled samples were subjected to a phase composition analysis using the XRD method. The recorded XRD diffraction patterns were used to determine the possible changes in the phase compositions and the calculation variation of the lattice parameter for Ni, YSZ, and GDC. Based on this data, the variation of cell volume for yttria-stabilized zirconia solid solutions, gadolina-doped ceria and metallic nickel were calculated. The results were compared with the data that was recorded for the initial sample Ni, YSZ, or GDC and the same sample that was heated without the solid fuel under the same conditions, i.e., at 850 °C for 100 h. This made it possible to obtain information regarding the changes that occur in the individual components of the cermets—Ni-YSZ or Ni-GDC—after prolonged contact with the tested solid fuels from series P0 to P850.

### 2.5. Analysis of CO Production during the Thermal Processes That Occurred in the Carbon Bed That Formed in an Anode Chamber of DC-SOFCs in the Temperature Range of 25–850 °C

Biomass-derived carbonaceous materials in the form of torrefied samples, biochars, or raw, ground pistachio shells were introduced to an anode chamber of the DC-SOFC (1) (a description of the DC-SOFC is included in the next section). A complete DC-SOFC was placed in an electric tube furnace and heated up to 850 °C. The ramp of the temperature increase was 5 °C/min. Nitrogen or CO_2_ gas was introduced into the anode chamber of DC-SOFC, and the flow rate of the N_2_ or CO_2_ gas was established to 20 cm^3^/min. Gas sampling for the chemical analyses was taken approximately 30 min after the temperature stabilized. The analyses of the main evolved gases as CO and CO_2_ from the anode chamber of the DC-SOFC at stabilized steady-state conditions were carried out in the temperature range 700–850 °C, increasing the temperature in steps of 50 °C. Gas samples from the anode chamber were taken using a laboratory syringe with clamps attached, and the gas samples of approximately 5 mL were injected onto a chromatography column. Thermo Scientific 1310 with a Seppack N column was used for the chemical analysis of the chemical composition of evolved gases. The tests aimed to obtain data regarding the variations in the content of the two main gaseous products (CO and CO_2_), which can be produced in the solid carbon bed that was placed in the anode chamber as a function of temperature. These tests reflected the variation of chemical composition of the gases due to the interaction between the surrounding gas atmosphere on a fixed carbon bed, which was placed in the DC-SOFC. These experiments provide useful information regarding the capability of applied solid carbon powders to produce CO or CO_2_ in the anode chamber, which can further influence the electrochemical performance of DC-SOFC. The experiments were performed when the DC-SOFC reached a stable open-circuit value (OCV).

### 2.6. Electrochemical Investigations of DC-SOFCs

The electrochemical oxidation of carbon particles was studied in two types of DC-SOFCs, which were marked as DC-SOFC (I) or DC-SOFC (II) and varied only in cathodic materials.

C|Ni -GDC|Ni-YSZ|YSZ|LSM-GDC|LSM|O_2_ (I)

C|Ni -GDC|Ni-YSZ|YSZ|LSCF-GDC|LSCF|O_2_ (II)

where YSZ is an electrolyte (8% mol Y_2_O_3_ in ZrO_2_) with a thickness of 150 μm; LSM is a La_0.8_Sr_0.2_MnO_3_ cathode material; LSM-GDC is a composite cathode material of 53 wt% La_0.8_Sr_0.2_MnO_3_ and 47 wt% Gd_0.10_Ce_0.90_O_1.95_; and Ni-YSZ is a cermet anode material composed of 50 vol% 8YSZ and 50 vol% Ni. Additionally, the (La_0.60_Sr_0.40_)_0.95_Co_0.20_Fe_0.80_O_3-x_ (LSCF) cathode material was used in case cell (II). Composite materials of (La_0.60_Sr_0.40_)_0.95_Co_0.20_Fe_0.80_O_3-x_ 52.4 wt% and 47.6 wt% Gd_0.10_Ce_0.90_O_1.95_ (GDC) were also used. The thickness of both the cathode and anode materials was ~50 μm. All electrode layers were prepared using the screen-printing method. The geometric area of the surfaces of the active electrode materials was ~1.60 cm^2^. The solid button oxide fuel cells were supplied by Fuel Cell Materials, United States of America (USA) [33]. Electrochemical measurements were carried out in a temperature range of 700–850 °C. The following two different gases were used in these investigations: N_2_ (purity 6N), which was introduced as a shielding gas to anode chamber of DC-SOFC (I) or (II); and CO_2_ (purity 6N), which was used as a gasification agent and was introduced to the anode chamber of DC-SOFC (I). The flow rate of the N_2_ or CO_2_ gas was established as 20 cm^3^/min. The electrochemical station with the potentiostat PGSTAT 300 N was used in the investigations. The procedure of experimental conditions that was applied to electrochemical investigations was described in our previous works [10,26,34] For selected solid fuels, including biochar P850, charcoal CH-M and Carbon Black; CB-221, the electrochemical curves and voltage U-current I were recorded in the humidified nitrogen. The humidified N_2_, which was by passing a gas stream through the scrubber, was then directed to the anode space.

## 3. Results

The total content of the elements, such as carbon, hydrogen, and sulfur, is one of the main factors that determines the possibility of using solid fuel to supply DC-SOFCs. Table 1 shows the results of the elemental analysis of carbon, hydrogen, and sulfur for raw, ground pistachio shells (P0), torrefied pistachio shells (P200, P300, and P400), and charred samples (P600 and P850) and the calculated amount of oxygen as the sum of oxygen and nitrogen. Additionally, the data for the content of ash and moisture that was determined for these samples was added.

The data in Table 1 demonstrates that increasing the temperature of the thermal treatment of ground pistachio shells from 200 °C to 850 °C results in a gradual increase of the total carbon content in all the solid carbonaceous fuels. Increasing the pyrolysis temperature from 600 °C to 850 °C does not cause significant changes to the total carbon content in the solid fuel samples. Regarding the P600 and P850 samples, the carbon content remains around 87–88%. Increasing the pyrolysis temperature of the pistachio shells leads to a gradual decrease of the hydrogen, oxygen and sulfur content. The hydrogen and oxygen are mainly removed as H_2_O from the samples. Moreover, a variation in the humidity content for the series of samples from P0 to P850 is observed. Although the applied thermal treatment of pistachio shells in higher temperatures led to decreased moisture, it did not completely remove it. This is probably due to the adsorption of moisture during the cooling samples when it was flowing through quartz reactor nitrogen as a shielding gas. Regarding combustion technology, a higher content of moisture decreases the calorific values of solid fuels. However, when solid carbon fuels in DC-SOFCs technology are applied, the phenomena can be different. A small amount of moisture can induce an additional gasification process in a solid carbon bed that is placed in DC-SOFC [1,35]. The increased temperature during the thermal treatment of pistachio shells caused the ash content to increase. When applying carbonaceous-based materials as solid carbon fuels in DC-SOFCs, a high content of carbon and low content of mineral matters in such fuels are desired. The main drawback of DC-SOFCs is the limited reaction zone of electrochemical oxidation of carbon particles, which corresponds to the direct contact with the surface of anode materials. The increased contamination of solid carbon fuels that are used in these types of fuel cells can reduce the reaction zone of the anodic oxidation of carbon particles [1,2]. Previous data that were reported in the literature and our previous works [1,35,36] showed that solid fuels involving more than 70% elemental carbon mass should lead to a good performance of the DC-SOFC. The presence of mineral content in solid carbon fuels is lower than 2–3% and does not cause the internal electrical resistance of fuel cells to increase significantly. The electrochemical performance of the DC-SOFC is expected to improve when inorganic elements that are included in mineral matter act as natural catalysts in the Boudouard reaction and improve production of CO in the anode chamber of DC-SOFC. Additionally, a low content of sulfur is required due to its negative impact on the Ni-GDC or Ni-YSZ anode structure [1,5,37]

The data obtained from the ultimate and technical analyses for the investigated samples from series P0 to P850 is sufficient to select these samples for further investigation as fuel to power the SOFCs.

**Figure 1:**
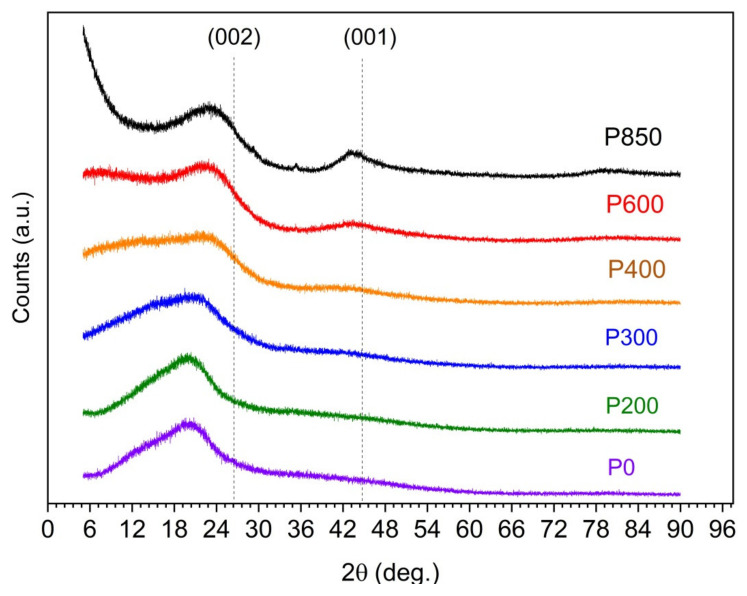
X-ray diffraction (XRD) patterns recorded for a series of carbonaceous materials prepared from waste pistachio shells (from P0 to P850).

Figure 1 illustrates the evolution of the XRD patterns that were recorded for the serial samples from P0 to P850 and reflects the variation in the structural ordering of the carbon particles of the solid fuels as a function of the applied temperature of the thermal treatment.

The diffraction patterns only show the weakly broadened peaks (002) and (001) that correspond to the partial graphitization of the samples. It should be emphasized that obtaining carbon samples with a disordered structure is a desirable feature due to their use as fuel for the utilization of DC-SOFCs.

As shown by the recorded XRD patterns, the initial P0 sample is characterized by a high background level with a strongly broadened peak (002) at 20–24°. A direct comparison of the recorded XRD patterns for the investigated sample series shows that the main peak in these patterns is mostly (002), which is typical for graphite-based carbon materials. The variation of the temperature preparation samples leads to differences regarding the intensity, width, and position of the (002) peak. The maximum diffraction reflection (002) shifts to higher angles than the typical graphite (002) position (~26.5 degrees). Additionally, the charred samples (P400, P600, and P850) that were prepared at the highest temperature showed a second peak (001), which is characteristic of the carbon-graphite structure. These results agree with the XRD studies that were carried out for other biomass types [17,23,26].

An XRD analysis was used to investigate the variation of the phase composition of the mineral residue (ash). The ash samples that were investigated came from the combustion of the P0–P850 origin series. The results revealed mineral residue, which might have undergone some variations in its chemical composition depending on the history of the previous thermal treatment in the temperature range of 200–850 °C and the subsequent combustion process at a temperature of 1000 °C.

Nevertheless, these experiments and results are useful to determine the influence of the chemical composition of inorganic compounds (especially alkaline oxides or iron-based oxides) on the gasification process of the carbon fixed solid bed, which was formed in the anode chamber of the DC-SOFC operation.

**Figure 2:**
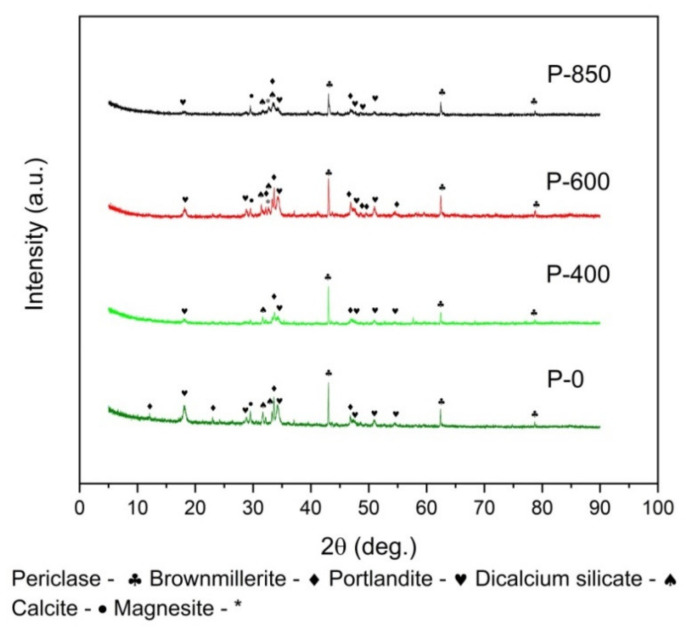
Evolution of XRD patterns that were recorded for inorganic compounds included in ashes. Ashes were prepared by burning the biochars P0, P400, P600, and P850.

As shown in Figure 2, the XRD pattern reflects the variation of the phase composition of the ash samples, which depends on the thermal history of the original samples (P0, P400, P600, and P850). The analyses that were performed on the variation of the composition phase of the ashes that were obtained after burning the samples showed that the main components of the ash that was obtained from the pistachio shells and burnt samples were as follows: Periclase (MgO), Calcite (CaCO_3_), and Magnesite (MgCO_3_). On the one hand, the individual inorganic phases that comprise the mineral residue can influence the operating parameters of the DC-SOFC. Within the operating temperatures (800–850 °C) of the DC-SOFC, decomposition of the alkali carbonates CaCO_3_ or MgCO_3_ to MgO and CO_2_ may occur [38,39]. The presence of CO_2_, MgO, and CaO can facilitate the solid fuel gasification process in the anode chamber in the DC-SOFC [40]. On the other hand, the presence of Ca_2_SiO_4_ and 4CaO·Al_2_O_3_·Fe_2_O_3_ oxides in the solid fuel can lead to an increase of the electric ohmic resistance of the fuel cell. Additionally, they can block the electrochemical reaction zone between the carbon particles and the surface of the cermet anode material where the electrochemical oxidation process takes place [41,42]. Additionally, an MIR was applied to study the chemical composition of the biomass of the original ashes.

**Figure 3:**
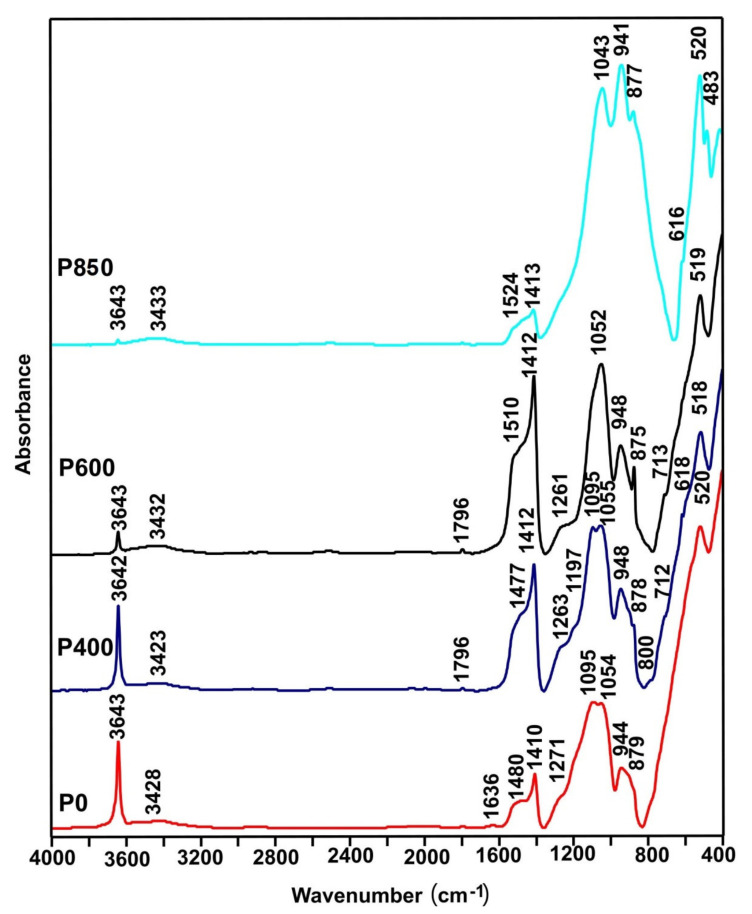
Midinfrared spectroscopy spectra recorded for ashes that were obtained from the P0 and P400–P850 ashes.

Figure 3 presents the collection of the MIR spectra that were recorded for the selected ashes that were obtained after combustion of the P0, P400, P600, and P850 samples.

All spectra presented in Figure 3 demonstrate a band at 3643 cm^−1^. This is characteristic of OH- groups (O-H stretching vibration) and occurred in the Portlandite (Ca[OH]_2_) [43]. Another peculiarity is the presence of bands at approximately 1410 cm^−1^, 875 cm^−1^, and 712 cm^−1^, which corresponds to the presence of CO_3_^2−^ groups. The location of the band at 712 cm^−1^ indicates the presence of calcite in the investigated ash samples [44]. In turn, the bands at approximately 940 cm^−1^ and 520 cm^−1^ (internal vibrations in [SiO4]^4−^) confirm the presence of dicalcium silicate in the samples. In addition to the bands mentioned above, all spectra show band(s) at approximately 1050 cm^−1^, which is typical for polymerized silicates [45]. An analysis of the MIR spectra presented in Figure 3 shows that as the temperature increases, the decomposition of portlandite progresses, which is indicated by a systematic decrease in the band intensity at 3643 cm^−1^. At 850 °C, a sharp decrease in the intensity of the bands that are associated with carbonate groups is characteristic, which indicates the decomposition of carbonates [46,47].

The Raman spectroscopy techniques determine the degree of disorder in the crystallographic structure of the tested series of samples in relation to traditional carbon materials (graphite, amorphous carbon, such as carbon black or other solid fuels made from waste biomass).

**Figure 4:**
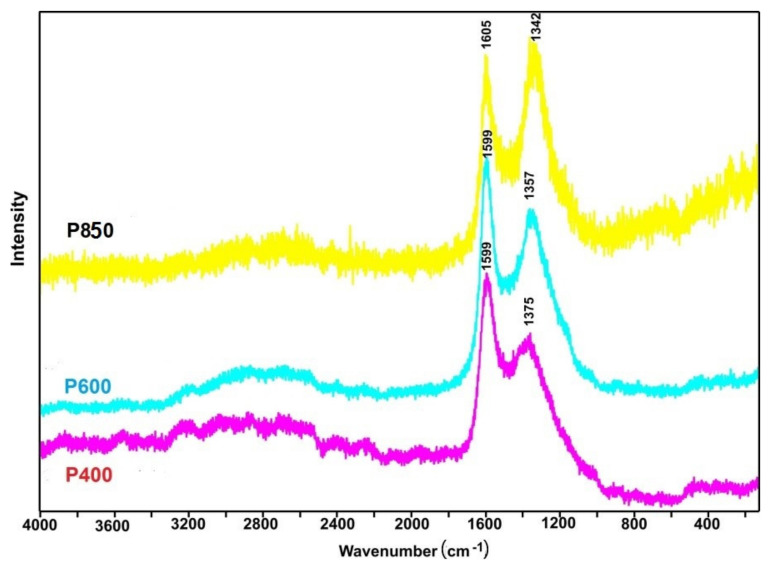
Raman spectra recorded for charred pistachio shells samples at temperatures of 400 °C, 600 °C, and 850 °C.

Figure 4 shows the Raman spectra for charred pistachio shells and samples P400–P850. Based on Raman research, it is relatively easy to determine the form of carbon that is present in the tested materials.

An analysis of the Raman spectra showed clearly visible G bands at approximately 1600 cm^−1^, which indicates the presence of carbon in the sp^2^ hybridization. The presence of D bands at the range of 1340–1375 cm^−1^ indicates the presence of numerous structural defects in the carbon phase [48]. The ratio of intensities of the G and D bands can be attributed to the degree of disorder of the carbon structure [49]. Additionally, the evolution of the D band with the increasing temperature of pyrolysis was observed from 1375 to 1342 cm^−1^.

It was previously determined that the presence of highly disordered carbon particles in solid fuel enhances the electrochemical oxidation of carbon particles in DC-SOFCs [24,26,50]. One key factor of diffusion and convection in the small solid carbon bed is the structure (i.e., the distribution of particle sizes, their compaction, and the surface area that is available for the medium to flow between and within the carbon particles)

Skrzypkiewicz and Antunes [51] examined the mechanism pathway in the direct carbon bed SOFC and its implications for the electrochemistry of such fuel cells. It was determined that the reaction mechanism at the anode is rate-limited by the electro-oxidation of the CO (anode), and the gas transport is mainly driven by natural convection (anode). The dominant process for the transport of CO, which can be electrochemically oxidized, is convection through the solid carbon bed. Knowledge of the evolution of the morphological structure of solid fuels produced from pistachio shells is necessary to explain the influence of the physicochemical properties of applied solid fuels on the performance of DC-SOFCs.

**Figure 5:**
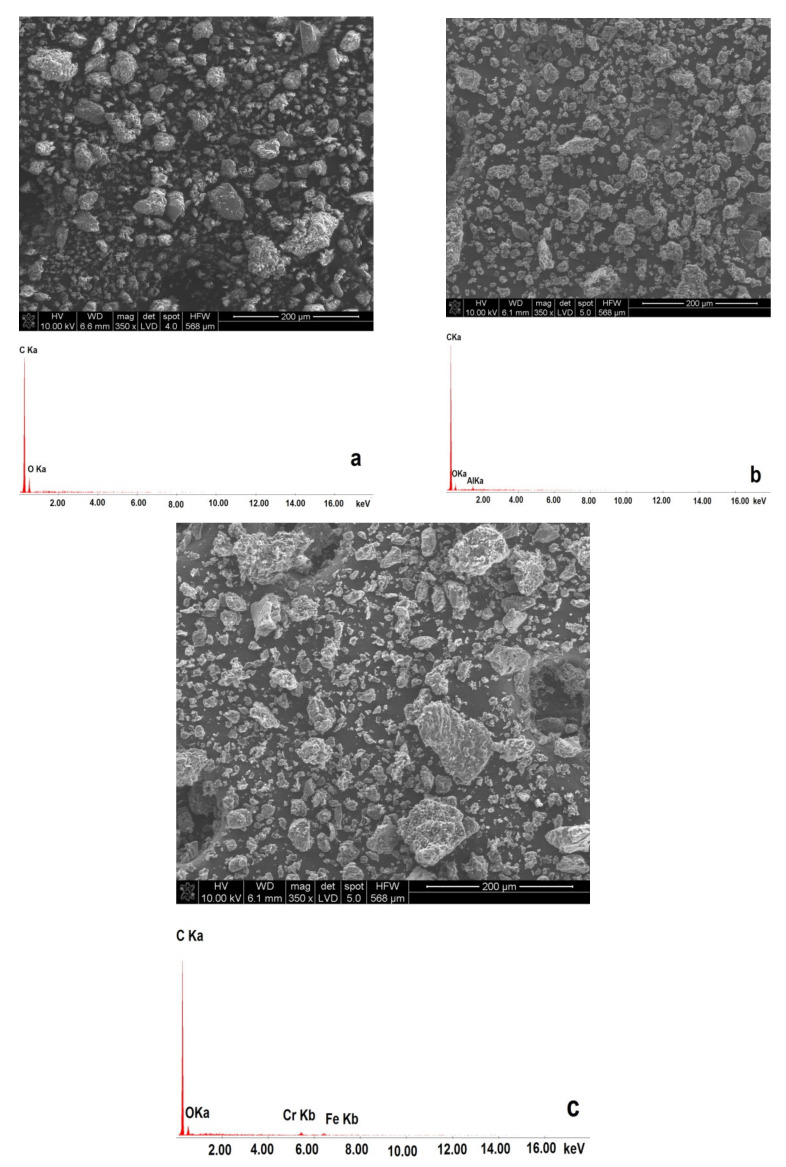
(**a**–**c**) SEM image of morphology for samples P400 (**a**), P600 (**b**), and P850 (**c**).

Figure 5a–c present the SEM images of carbonaceous materials prepared from pistachio shells.

**Figure 6:**
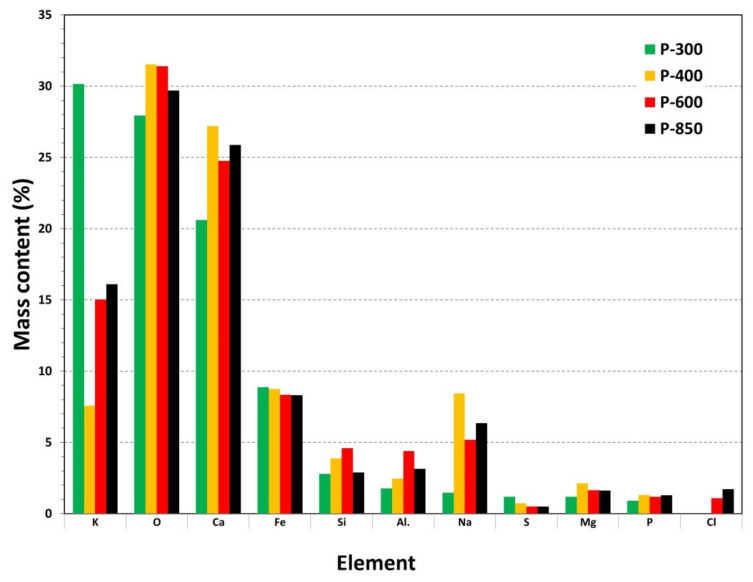
Results of comparative qualitative chemical analysis for solid fuel samples P400, P600, and P850, which are derived from pistachio shells. Mass content of the elements was determined using WDXRF method.

The analysis of the SEM images from Figure 5a–c shows that increasing the temperature during the thermal treatment of pistachio shells results in a slight increase in the average grain size. Observing the morphology of grains shows that they are more isometric in shape. Desalux et al. [49] investigated the impact of the morphology of carbon particles on the anodic oxidation of solid fuels in DC-SOFC. They found that irregular shapes of carbon particles in solid fuels led to “only point contact” with surface anode electrodes. This phenomenon reduced the limited electrochemical oxidation of the carbon particles’ reaction zone. Additionally, a higher temperature of above 400 °C increased porosity in the grains of prepared solid fuels. During the SEM observation, a qualitative analysis of the samples was performed. Although the grains of solid carbon fuels derived from pistachio shells mainly consisted of carbon, small amounts of Al, Fe, Cr were detected [21,52]. Wavelength Dispersive X-ray Fluorescence (WDXRF) was used as a second method for a rapid qualitative chemical analysis of solid carbonaceous samples [53,54]. The main metallic elements that were detected were K, Mg, Ca, Zn, Sr, and Fe, and some traces of P and Cl was visible. Inorganic content can vary depending on the biomass types, as thermal treatment affects the variation of concentrations of elements and compounds. This study focused on the semi-quantitative comparative analysis of the main elements, such as K, Mg, Ca, Sr and Fe, which can vary depending on the temperature of the prepared samples. Data in review papers that analyzed the influence of factors on the efficiency of solid carbon fuels gasified with CO_2_ as medium indicated that these elements improve the kinetics of the Boudouard reaction during the carbon gasification process, especially in the temperature range of 700–850 °C [55]. The comparative qualitative analysis that was performed for the solid fuel samples P300, P400, P600, and P850, which are derived from pistachio shells, is presented in Figure 6.

The analysis of the comparative variation between the mass content of Fe or alkaline elements, focusing on K, Na, Ca, and Mg for carbonaceous-based materials from the series P300 to P850, found no considerable differences in the chemical composition due to the applied thermal treatment of pistachio shells from 300 °C to 850 °C. The difference in the content of K and Na is obvious in the case of sample P400, probably because of the inhomogeneity of P400 samples, which can happen when applying thermal treatment at 400 °C for 2 h. At this temperature, a high number of gaseous products and liquid compounds are removed. This statement agrees with the DTA curve recorded for the P400 sample. A small exothermic effect at ~415 °C is visible, which may correlate with the decomposition of potassium salts. The data that were obtained using the WDXRF method are in good agreement with the observed changes of the phase composition recorded using the XRD method for the ashes (Figure 2) and the MIR investigations for the solid carbonaceous samples (P400 to P850) (Figure 3). Fe_2_O_3_ catalyzes the reverse Boudouard reaction according to chemical Equations (5) and (6) as follows [55]:

Fe_m_O_n_ + CO_2_→Fe_m_O_n+1_ + CO (5)

Fe_m_O_n+1_ + C→Fe_m_O_n+1_ + CO (6)

Tan and You [56] confirmed the usefulness of the Fe-load charcoal samples as fuels for DC-SOFCs. The power output of the investigated DC-SOFC was significantly improved compared to that of the same DC-SOFC that was supplied by charcoal without the addition of the Fe_2_O_3_ catalyst. Skrzypkiewicz et al. [57] investigated the impact of Fe_2_O_3_-loaded charcoal on the performance of DC-SOFC. The reverse Boudouard reaction (C + CO_2_→2CO) is a limiting process for the fuel cell performance. The in-situ-generated CO improved the performance of DC-SOFC compared to that of the same cells that were supplied charcoal without the Fe_2_O_3_ catalyst.

Wu et al. [19] investigated the performance of DC-SOFC when involving the internal catalytic gasification of carbon to gaseous carbon monoxide via the reverse Boudouard reaction (C[s] + CO2[g]→2CO[g]). The carbon gasification reaction rate was greatly enhanced when Fe_m_O_n_–M_x_O (M = Li, K, Ca) was introduced as a catalyst for solid carbon fuels. They proposed that the following reactions are responsible for improving the gasification and performance of the DC-SOFC:

(Li_2_,Ca)CO_3_ + C→(Li_2_,Ca)O + 2CO (7)

(Li_2_,Ca)O + CO_2_→(Li_2_,Ca)CO_3_ (8)

The above inorganic compounds are natural components of the series of biomass-derived solid fuels (P300 to P850).

Using the obtained carbonaceous-based materials as a solid fuel to power SOFCs requires an understanding of the thermal effects that can occur in powdered solid fuels that are placed in an anode chamber of the DC-SOFC. The temperature range in which a DC-SOFC operates is large. It includes the following stages: (i) turning on the DC-SOFC at RT temperature; (ii) heating it up to the operating temperature of the cell (700–850 °C); and (iii) targeting the operation under stabilized conditions (700–850 °C). Changes in the physicochemical properties of solid fuels with temperature variations influences the DC-SOFC operation, as this can affect the durability of the anode and electrolyte materials and the achieved operating parameters (i.e., the current and power densities derived from the DC-SOFC).

Pistachio shells are assumed to be composed of hemicellulose, cellulose, and lignin, and products of thermal decomposition can also influence the performance of DC-SOFC.

**Figure 7:**
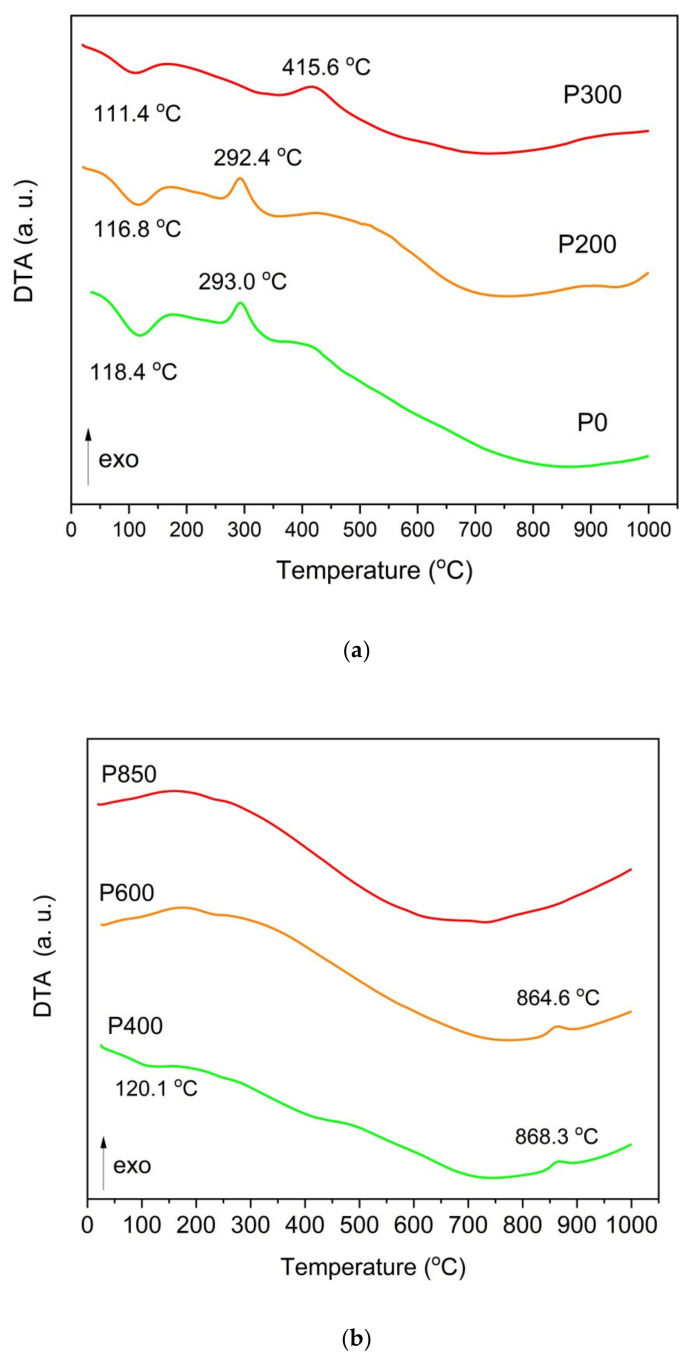
(**a**) DTA curves recorded for ground raw pistachio shells (P0) and torrefied shells (P200 and P300). N_2_ is used as a shielding gas. (**b**) DTA curves recorded for charred pistachio shells P400, P600, and P850. N_2_ is used as a shielding gas.

Figure 7a,b record the DTA curves for ground raw pistachio shells, torrefied shells, (Figure 7a) and chars that are prepared at temperatures of 25–1000 °C (Figure 7b).

**Figure 8:**
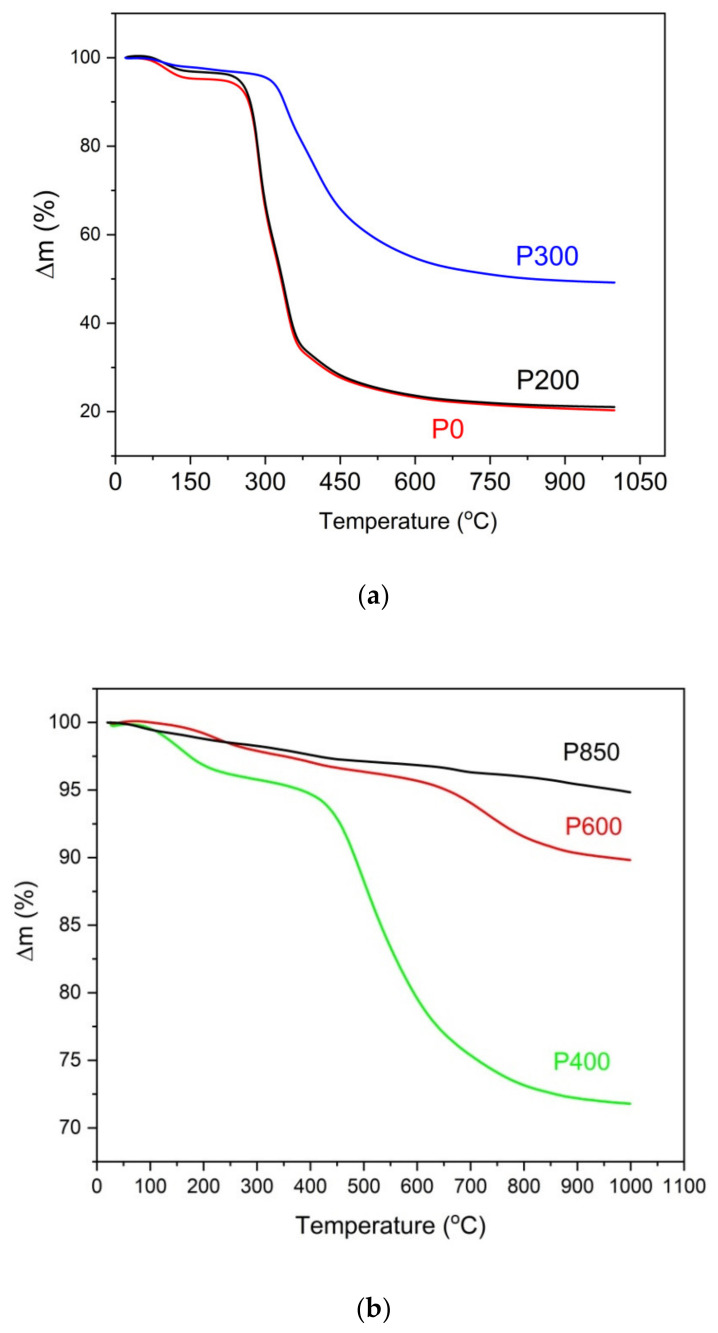
(**a**) TG curves recorded for ground raw pistachio shells (P0) and torrefied shells (P200 and P300) parallel to DTA investigations (Figure 7a). N_2_ is used as a shielding gas. (**b**) TG curves recorded for charred pistachio shells (P400, P600, and P850) parallel to DTA investigations (Figure 7b). N_2_ is used as a shielding gas.

As shown in Figure 7a, gradually heating these samples resulted in an initial slight thermal effect at a temperature of approximately 100 °C, which is a result of the water evaporating from the sample. Heating the tested samples between the temperature range of 100–350 °C initiates the pyrolysis processes related to the first decomposition of organic matter, which occurs between 150–350 °C and 275–350 °C, respectively, for hemicellulose and cellulose. The lignin decomposition occurred at higher temperatures between 275–500 °C. In the DTA curves for the solid fuel samples (P400–P850, which are derived from pistachio shells) (Figure 7b), some thermal effects corresponded to the decomposition of inorganic compounds at 840–850 °C. The wide exothermic peak that is visible on the DTA curve is likely connected to the decomposition of calcium carbonate (CaCO_3_). Figure 8a,b show the registered TG curves vs. the temperature that was recorded for the raw samples (P0), torrefied pistachio samples (P200 and P300) (Figure 8a) and charred samples (P400, P600, and P850) (Figure 8b).

The analysis of the TG curves that were recorded for the studied pistachio shells show that the increased temperature of the thermal treatment of the pistachio shells led to decreased mass losses in the N_2_ gas atmosphere. The biochar is formed from pistachio shells at approximately 600 °C. When the temperature of the thermal treatment of pistachio shells is higher than 600 °C, it decreases the sample mass due to possible surface oxidation of carbon or gasification carbon samples. The observed mass losses for samples P600 and P850 are considerably lower than for sample P400.

**Figure 9:**
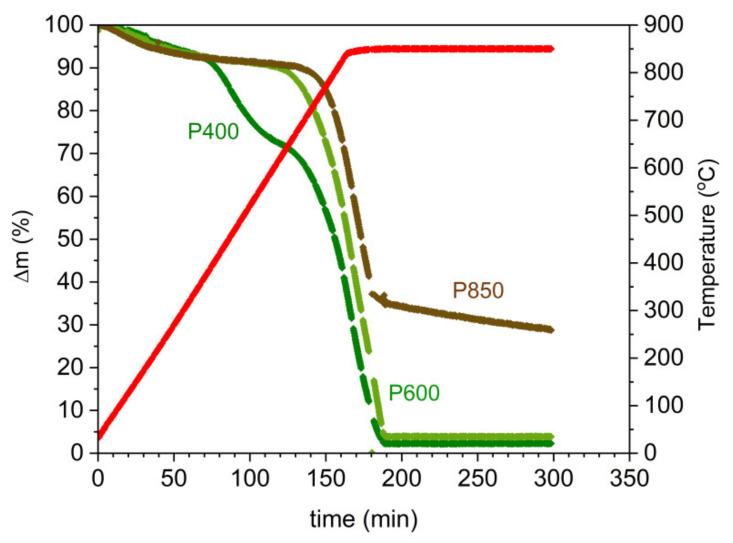
TG curves vs. time recorded for temperatures of 25–850 °C for samples P400, P600, and P850. Measurements were performed in a CO_2_ gas atmosphere using a thermobalance.

The conditions of the solid fuel preparation reflect the efficiency of the gasification process of solid carbon via a CO_2_ gas medium, which is introduced through an external source. Knowledge about the progress of the gasification reaction of solid carbon to carbon monoxide via CO_2_ as a gasification agent reaction (3) is important for the practical application of prepared solid fuels in DC-SOFC technology [58]. In this study, the TG curves using a thermobalance registered the variation of mass losses vs. the temperature in nonisothermal and isothermal conditions at a temperature of 850 °C vs. time. The registered mass losses vs. the temperature and time are shown in Figure 9.

Based on the plotted dependence of the thermogravimetric curves Δm = f (T) for the samples P400, P600, and P850, the following two characteristic stages can be distinguished: (i) the heating of the sample from 20 °C to 850 °C with a temperature increase of 5 °C/min, where the duration of the first stage is approximately 200 min; and (ii) isothermal, i.e., heating the tested materials at 850 °C until a constant mass is reached.

The analysis of the dependence Δm = f (T) for the samples P400, P600, and P850 determined two temperature ranges, in which the pyrolysis and gasification processes occurred. Table 2 presents the data of the estimated temperature ranges for the first and second stages and the loss of mass Δm of the samples P400 to P850 during the pyrolysis and gasification processes.

The data in Table 2 show that the greatest weight loss in the gas atmosphere of CO_2_ in the first temperature stage (200–640 °C) is observed for the P400 sample, and the lowest is observed for the P850 sample. One of the reasons for increasing the gasification temperature of the P850 char sample compared to the P600 sample is the higher degree of carbon graphitization. The order of the carbon structure in sample P850 led to a decrease of many surface defects and active centers, which are desired for carbon reactivity towards CO_2_. The SEM observations demonstrated that the grain size of the solid fuel increases along with the increase in the preparation temperature of the chars. The increased grain sizes of the solid carbon fuels can postpone the temperature of the gasification process to a higher temperature range. Changes in the dispersion area of inorganic compounds, which are considered natural catalysts, also exhibited a limited catalytic effect compared to the prepared samples in lower temperatures. The analysis of the variation of mass losses Δm vs. temperature or time shows that the obtained solid fuels exhibited high reactivity in the CO_2_ gas atmosphere. Based on this data, it is expected that the CO that is produced from the gasification reaction C + CO_2_→2CO can improve the performance of the DC-SOFC at a temperature range of 750–850 °C.

**Figure 10:**
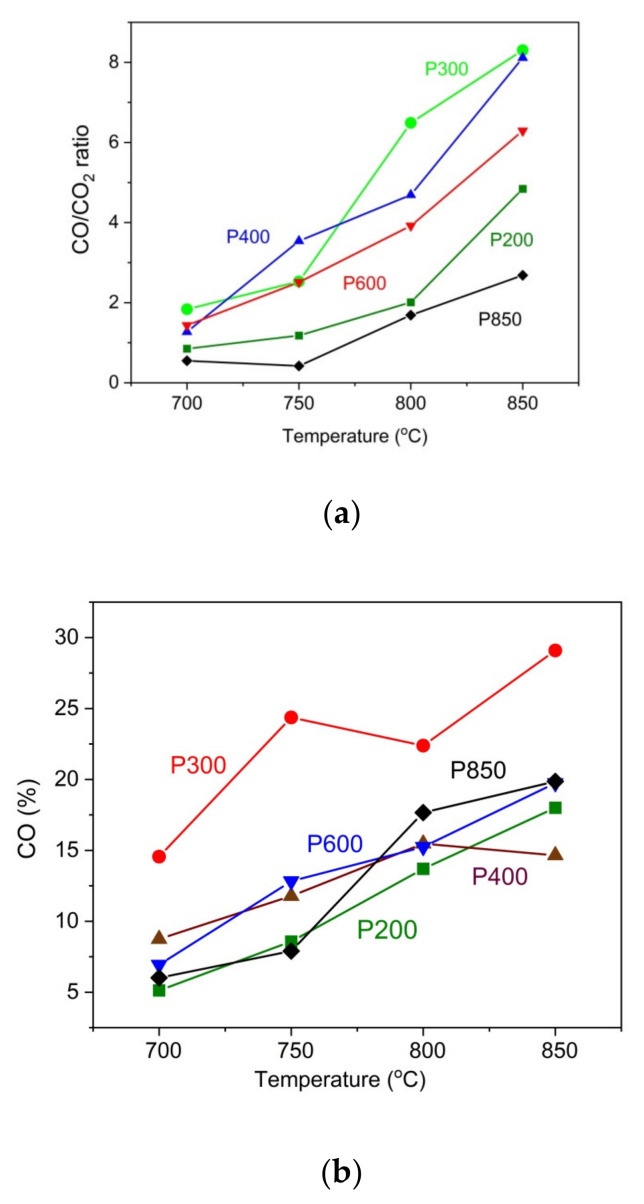
(**a**) Variation of CO/CO_2_ in outlet gases from anode from DC-SOFC (I) vs. temperature that was established in a solid carbon bed. Measurements were performed when N_2_ was introduced (20 mL/min) as a shielding gas to anode chamber of DC-SOFC (I). (**b**) Variation of CO in outlet gases from anode of DC-SOFC (I) vs. temperature that was established in a solid carbon bed. Measurements were performed when CO_2_ was introduced (20 mL/min) as a gasification agent to anode chamber of DC-SOFC (I).

Additionally, the possible production of carbon according to the reaction C + CO_2_→2CO in the anode chamber of DC-SOFC is considered. In Figure 10a, the variation of the main evolved gases as CO and CO_2_ from the anode chamber of DC-SOFC is presented compared to the temperature that was established in a solid carbon bed.

The data in Figure 10a show that the ratio of CO to CO_2_ increases as the temperature rises. In general, the increase in temperature improves the chemical kinetics of the Boudouard reaction, and a larger amount CO is produced. Moreover, the influence of the thermal treatment of pistachio shells on the CO/CO_2_ ratio variation is considerable. The highest CO/CO_2_ ratio in these experimental conditions was recorded for the P300 and P400 samples. The lowest CO/CO_2_ ratio was observed for the P850 biochar and the P200 sample. Regarding the pistachio samples that were prepared at a temperature range of 300–400 °C, the observed increase of evolved gas products as CO and CO_2_ is a direct consequence of the thermal decomposition of residual organic carbon compounds, which originated from gradually degraded cellulose, hemicellulose and lignin in an inert gas atmosphere. The lowest concentration of CO/CO_2_ in the evolved gases was found in the biochar P850 sample, which was fuel-constituted solid carbon fuel. Therefore, the production of CO and CO_2_ is possible due to the partial oxidation of carbon particles.

Figure 10b presents a positive correlation between the amount of CO in the gas outlet of the anode chamber from DC-SOFC (I) and the temperature of the fixed carbon solid bed. This is a direct consequence of the Boudouard reaction C+CO_2_→2CO (4). In these experimental conditions, the CO_2_ from an external source is supplied to solid carbon powders and placed on the anode surface of the DC-SOFC. One result of this reaction is an increase in the amount of CO in the anode chamber. The highest reactivity was observed in the P300 sample, whereas the lowest reactivity was observed in the P200 sample. Regarding the charred samples (P850 and P600), a similar range of evolved CO from the anode chamber was observed at temperatures of 800 °C and 850 °C.

The pistachio shells that were charred at a temperature of 850 °C were selected for gasification investigations in a steam gas atmosphere. Knowledge of producing CO, H_2_, and CH_4_ is important for practical applications of such carbonaceous-based materials in the DC-SOFC [58,59,60]. The gasification reaction of carbon particles with water vapor as a medium may result in the formation of further gaseous products, such as H_2_, CO, and CH_4_, which are valuable fuels for the DC-SOFC. Knowledge of the formation of such fuels in the anode chamber during the reactivity of H_2_O with carbon particles will be helpful for understanding that the power output of DC-SOFCs is supplied by different solid carbon fuels and the performance of DC-SOFCs in different experimental conditions. This includes when the humidified gas medium as N_2_ or CO_2_ is introduced to the anode chamber of the DC-SOFC to induce the gasification process and improve the performance of the DC-SOFC.

As the data in the literature that corresponds to an analysis of solid carbon fuels’ reactivity with H_2_O are limited, it is difficult to analyze how humidifying gases impacts the DC-SOFC’s performance.

The efficiency of the chemical reactivity of solid carbon particles under the gasification process can be expressed using the following main Equation (9):

dV/dt = f (t)(9)

where dVi/dt equals the rate of formation of a given product in cm^3^/min∙g, and t equals time in minutes.

These dependencies (dV/dt) were determined by measuring the concentration of gaseous products in the post-reaction gas, the flow of which was maintained at a constant level during the entire measurement. The release rate of a given product over time can be calculated using the following formula (10):

(dV_i_)/dt = V c_i_(t) (10)

where V̇ equals the volume flow of the reaction gas in cm^3^/min and c_i_(t) equals the concentration of a given product in the postreaction gas at time (t) in the percentage of volume. Based on the composition of the resulting gas, the curves of the generation rates of CO, CO_2_, H_2_ and CH_4_ vs. time are presented for the charred pistachio sample (P850) and commercial charcoal, which was utilized in the study as the reference material. During the first stage of the process, relatively high rates of gas generation were observed in pyrolysis using charcoal. During the second stage, the proper gasification of the resulting char was characterized by lower generation rates of the examined gases. Regarding the P850 sample, the shape of the curve differed to the curve of the commercial charcoal. During the initial stage of the process, relatively lower peaks were observed.

Later, a continuous increase in the gaseous generation rates of H_2_, CO, and CO_2_ was observed, which slowly decreased after reaching a peak. H_2_ is characterized as having the highest generation rate in both investigated samples. The generation curves of CO and CO_2_ are diverse and depend on the sample type.

**Figure 11:**
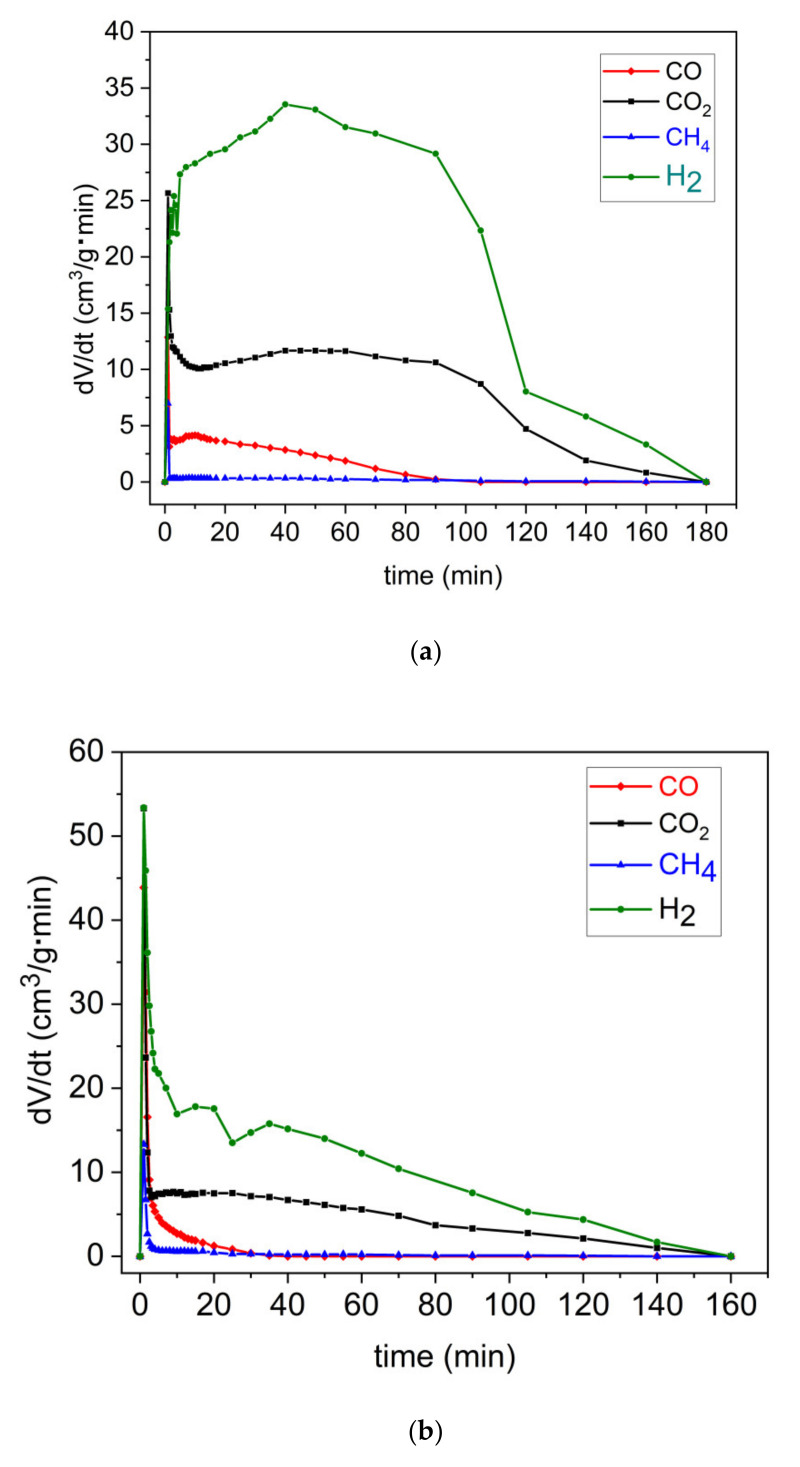

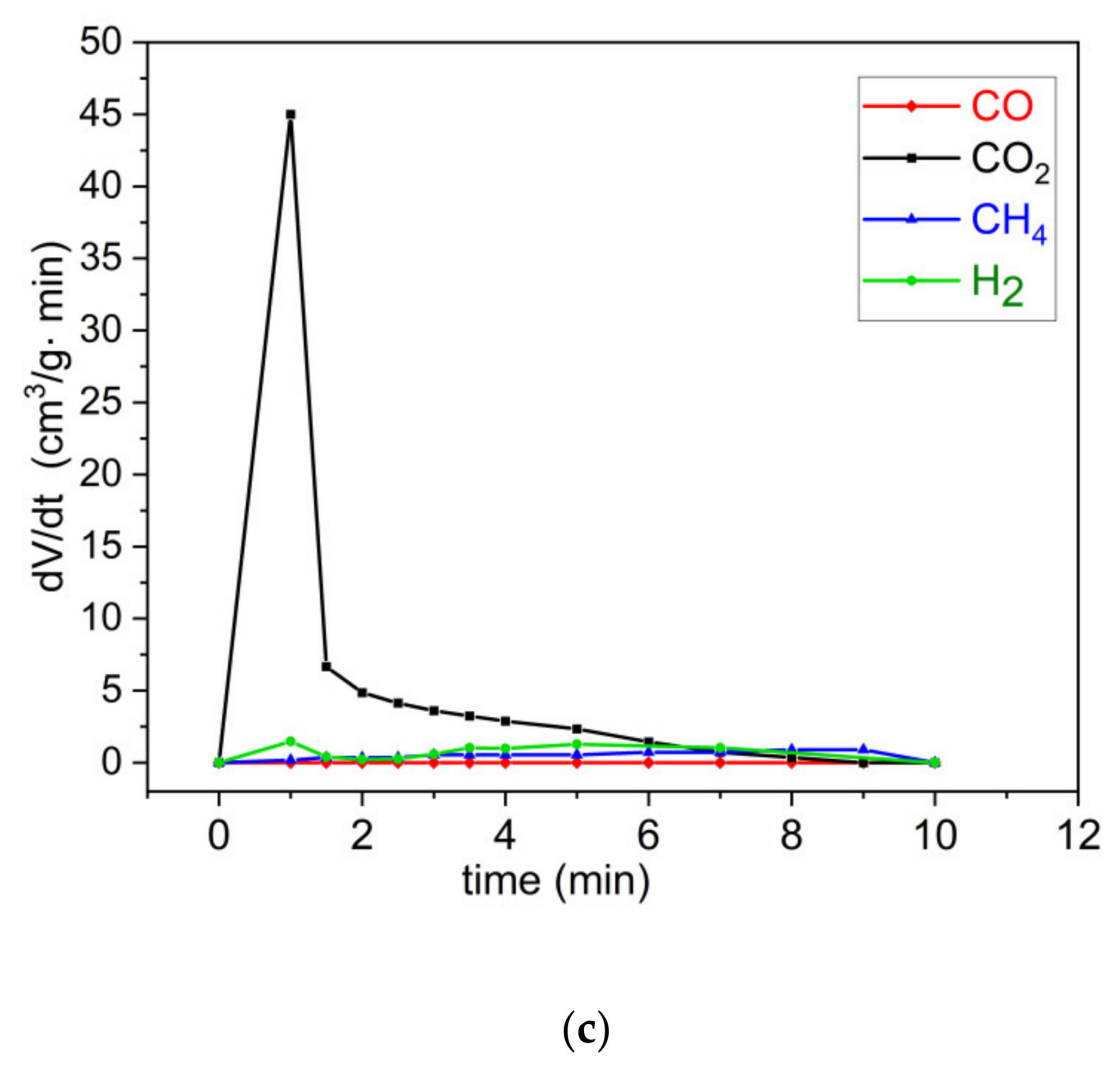
(**a**) Curves of evolution rate of gaseous products (CO, CO_2_, H_4_, and H_2_) of charred pistachio sample P850 at a temperature of 850 °C, as determined by volumetric method. (**b**) Curves of evolution rate of gaseous products (CO, CO_2_, H_4_, and H_2_) of Charcoal CH-M (Merck, Darmstadt, Germany) at a temperature of 850 °C, as determined by volumetric method. (**c**) Curves of evolution rate of gaseous products (CO, CO_2_, H_4_, and H_2_) of Carbon Black CB-221 at a temperature of 850 °C, as determined by volumetric method.

Figure 11a illustrates the main product of the gasification reaction of the analyzed biochar P850. The highest generation rate was H_2_, followed by CO_2_ and CO, respectively. Methane was only generated during the first few minutes of the gasification of the samples due to the simultaneously occurring residual pyrolysis reaction from the biochar. During the gasification of the char samples, the formation of methane was not observed. The generation rates of H_2_, CO_2_, and CO increased rapidly during the first few minutes of the gasification process. This was followed by a slight increase in the generation rates of both H_2_ and CO_2_ (up to 40 min) and then a decrease. The generation rate of CO decreased steadily after reaching a peak during the first few minutes of the process. At approximately 90 min, the CO generation reaction was negligible. From that moment, the only observed gaseous reactions were H_2_ and CO_2_, which were generated until the end of the process, i.e., up to the 180th min. Based on the analysis of gasification efficiency, the sample P850 was more reactive in the H_2_O gas atmosphere. Moreover, a higher generation rate of gaseous products, such as H_2_ and CO, was observed with the P850 sample. However, regarding methane, a slightly higher rate of CO_2_ generation was noticed in the commercial charcoal sample CH-M. Contrary to the previous samples, the Carbon Black (CB-221) exhibited very low reactivity when steam was used as a gasification agent.

The effectiveness of the carbon conversion rate from the P850 charred sample; charcoal CH-M or carbon black CB-221 to the gaseous products (CO, CO_2_, CH_4_, and H_2_) in the presence of a gasification agent (water vapor) is determined based on previous data presented in Figure 11a–c.

**Figure 12:**
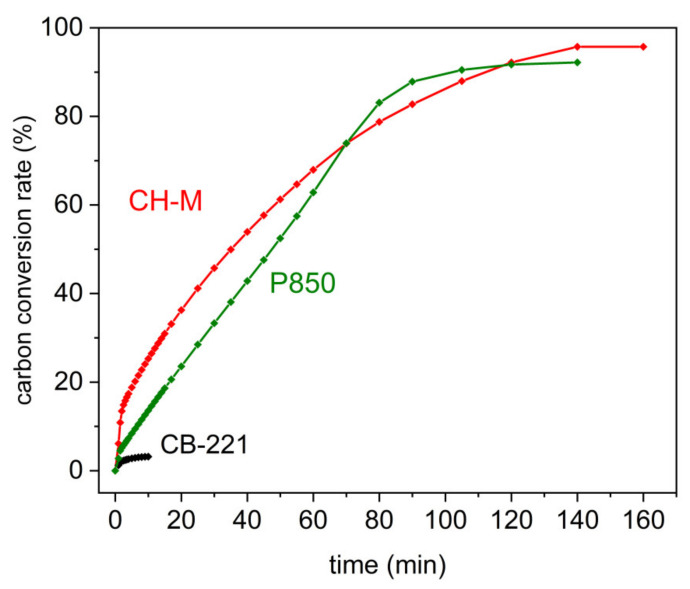
Carbon conversion rate from P850 sample charcoal CH-M, and Carbon Black CB-221 vs. time. Experimental conditions reflect those presented in Figure 11a–c.

Figure 12 indicates that the conversion of the solid fuels P850 or charcoal CH-M to gaseous products is comparable and more than 90%. The lowest total conversion rate was noticed for the Carbon Black CB-221 sample. Based on the research, it can be concluded that biomass-derived solid fuels can be easily converted to gaseous products in a humidified gas atmosphere. However, regarding carbon black sample, this amount is minimal. It also confirms that regarding biomass-derived solid fuels, the impact of gaseous fuel, such as CO, H_2_, and CH_4_, is more predictable than when applying carbon black as a solid fuel to supply the DC-SOFC.

### 3.1. Chemical Stability of Ni-YSZ or Ni-GDC Anode Materials in a Fixed Carbon Bed

Cermet composite materials containing Ni-YSZ and Ni-GDC are anode materials that are commonly used to construct a DC-SOFC. The anode material should exhibit significant chemical durability against the solid fuels that are used. In a DC-SOFC anode chamber, chemical reactions occur between the components of the anode material, carbonaceous-based materials, and a chemical gas atmosphere [60,61,62]. Issues related to the possible destructive effect of biomass-based fuels used in the DC-SOFC cell and the analysis of the problem of carbide formation from Ni-C and related binary systems were presented and discussed in a previous article [60]. Despite numerous attempts, this problem is not yet fully investigated and explained.

**Figure 13:**
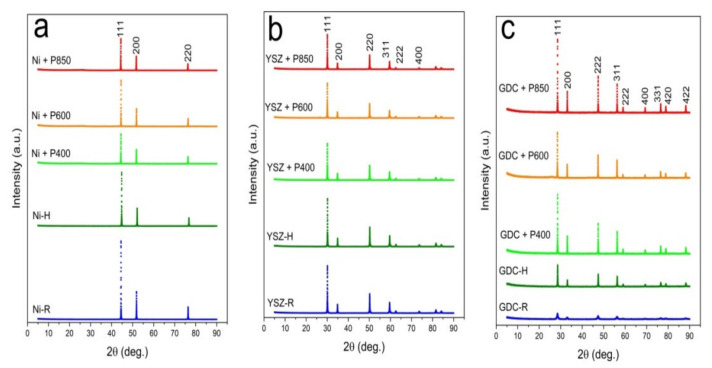
(**a**–**c**) XRD diffraction patterns for initial powder samples (marked as R) of Ni (**a**), 8YSZ (**b**), 20GDC (**c**) and same samples were additionally subjected to heating process at temperatures of 400 °C, 600 °C, and 850 °C for 100 h, respectively, without contact with solid carbon fuels (marked H), as were a series of mixtures with carbonaceous materials that were obtained from pistachio shells.

Figure 13a–c present the recorded diffraction patterns for the initial powder samples of Ni-R (a), YSZ-R (b), and GDC-R (c), powders Ni-H, YSZ-H, and GDC-H, when they were heated at 850 °C for 100 h without contact with solid carbon fuels, as were the mixtures of YSZ, GDC, and Ni samples with the appropriate carbonized products from samples P-400, P-600 and P-850. The initial samples of Ni, GDC, and YSZ in the XRD patterns are marked as R, and the same samples were additionally subjected to the heating process at temperatures of 400 °C, 600 °C, and 850 °C for 100 h, respectively, without contact with solid carbon fuels, which are marked as H.

**Figure 14:**
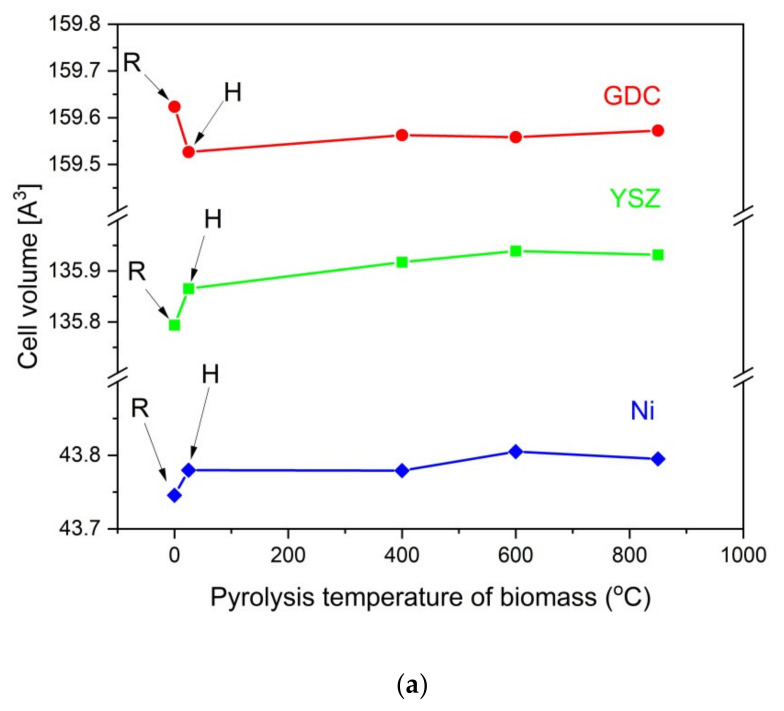

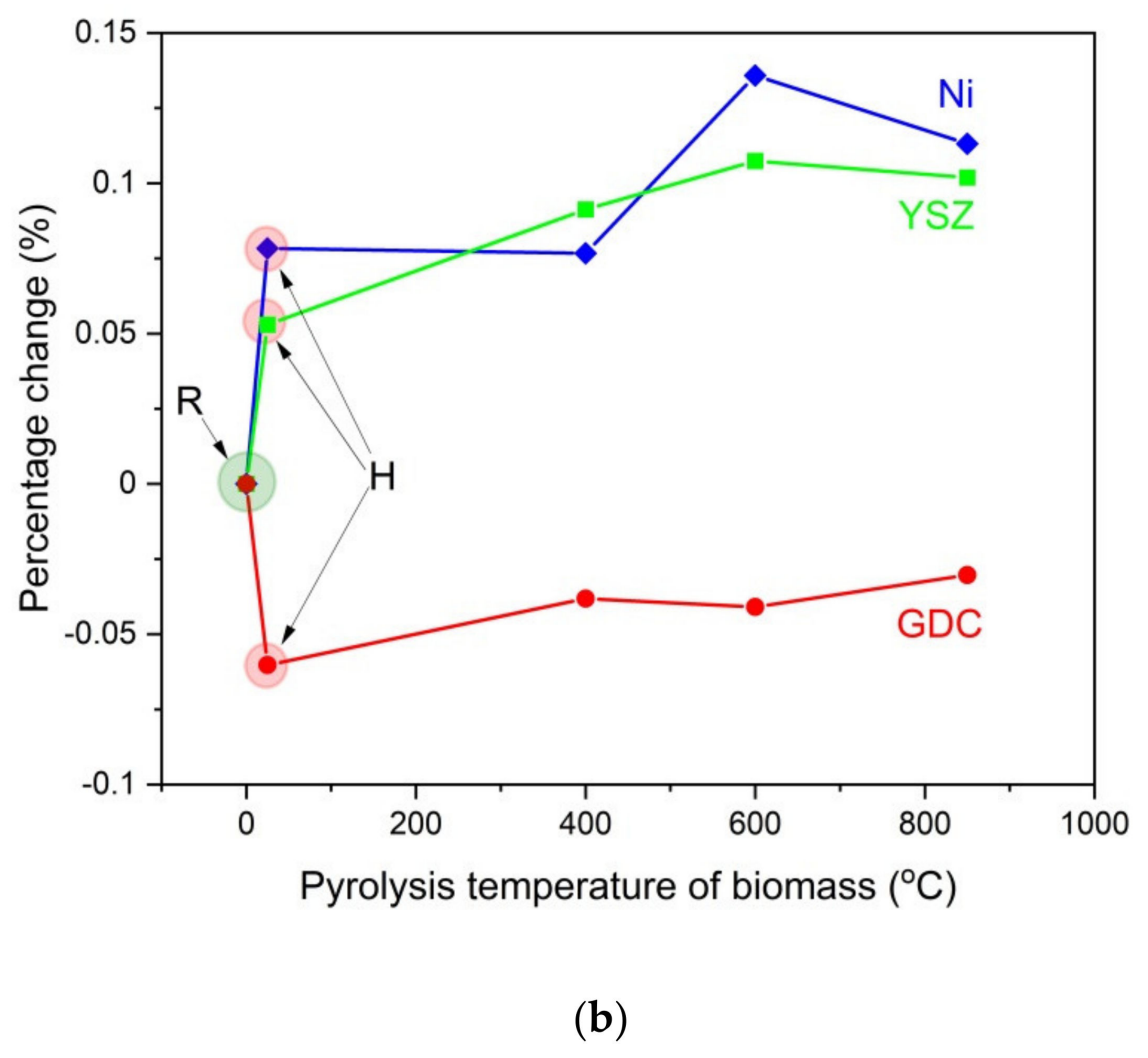
(**a**) Variation in calculated cell volume between base YSZ, GDC, and Ni samples (R) and after heating samples at 850 °C for 100 h without contact with solid carbon fuels (H) and after heating following mixtures: YSZ and solid fuels; GDC and solid fuels, and Ni and solid fuels. (**b**) Variation of percentage changes in cell volume for base YSZ, GDC, and Ni samples (R) after heating samples at 850 °C for 100 h without contact with solid fuels (H) and after heating following biomass mixtures: YSZ and solid fuels; GDC and solid fuels; and Ni and solid fuels.

Based on the recorded XRD patterns, variations in the unit cell volume were calculated for the base Ni, YSZ, and GDC samples before after being heated at 850 °C for 100 h without contact with solid fuels, as were a series of mixtures with carbonaceous materials that were obtained from pistachio shells. These data are presented in Figure 14a,b.

Individual analyses of cell volume variations for the base Ni, GDC, and YSZ samples (R) after the heat treatment without adding solid carbon fuels (H) were completed first. It was found that additional heating of each component led to a minimal increase (<0.05%) in the cell volume of Ni or YSZ. In the GDC sample, a decrease in cell volume was observed. Additional heating of the YSZ and GDC samples or allowing Ni to have direct contact with the carbon particles did not lead to significant changes in the cell volume of these materials. The greatest change in the cell volume was visible in the metallic Ni, which is possibly due to the diffusion of carbon into Ni particles. This phenomenon can be attributed to the dissolution of carbon particles in the metallic nickel structure. These results agree with the data that was analyzed for the chemical stability of Ni-YSZ or Ni-GDC anode materials with commercial charcoals, for instance Charcoal CH-M, which is described in this paper as reference material for charred pistachio shells (P850) [60].

### 3.2. Electrochemical Performance of SOFCs Powered by Solid Fuels from Pistachio Shells

**Figure 15:**
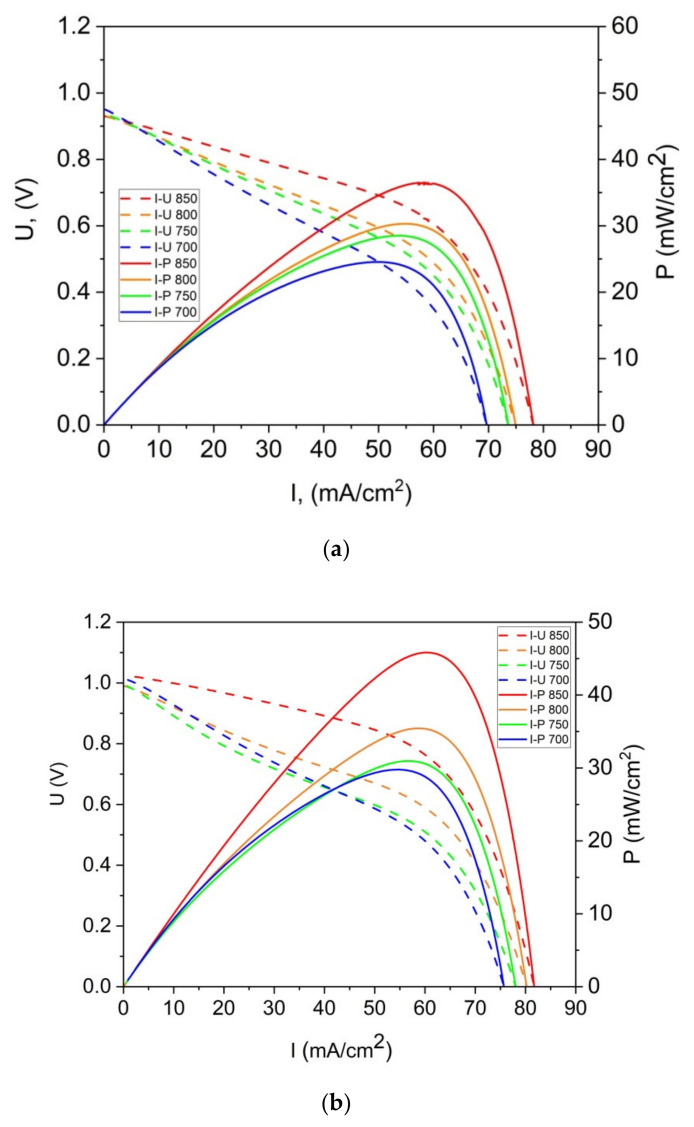

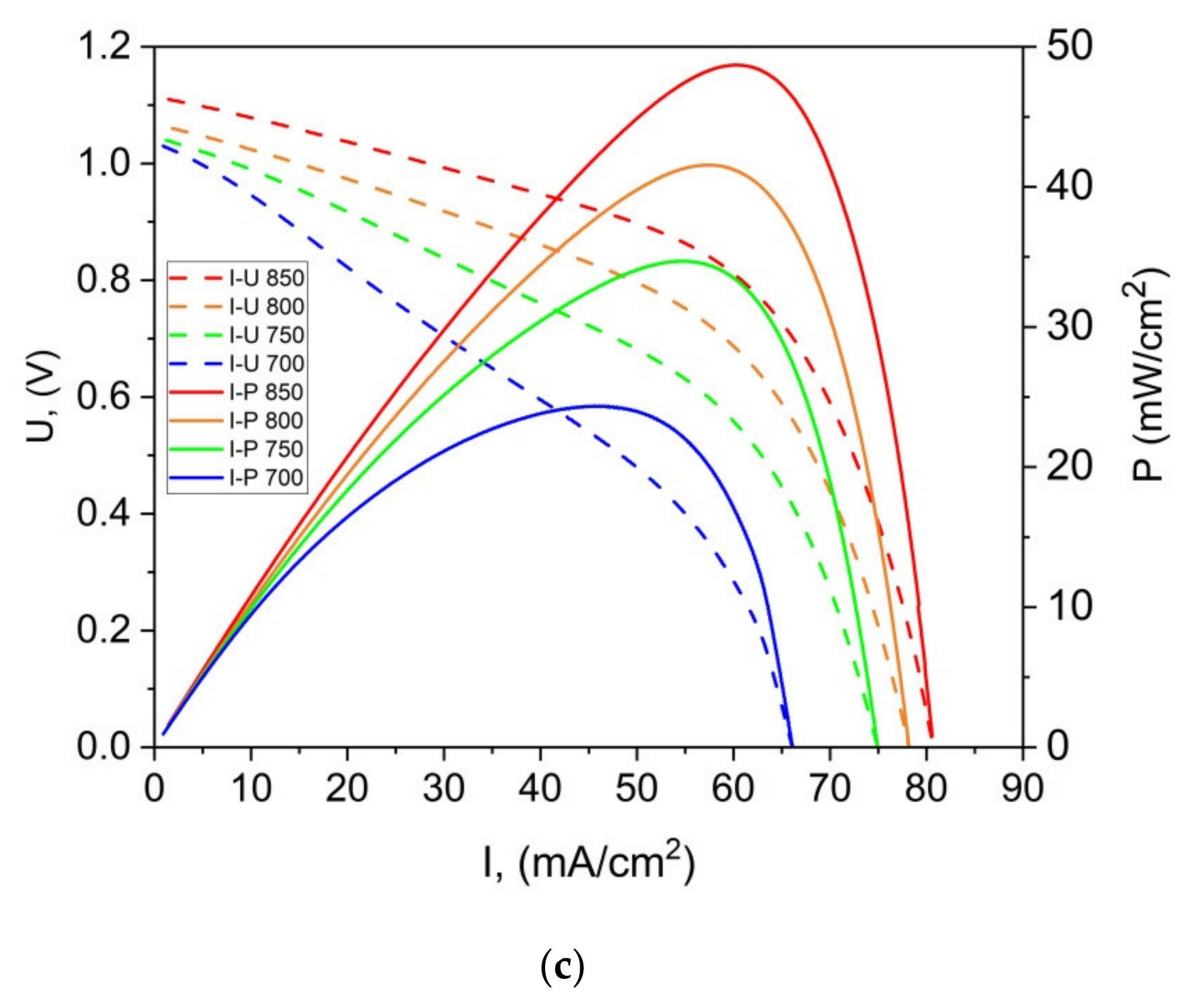
(**a**) Families of dependencies: voltage U–current density I and power density P vs. current density I, as determined for DC-SOFC (I) with a lanthanum-strontium-manganite (LSM) cathode and N_2_ atmosphere over a solid fuel (P0). Temperature range was 700–850 °C. (**b**) Families of dependencies: voltage U-current density I and power density P vs. current density I, as determined for DC-SOFC (I) with LSM cathode and N_2_ atmosphere over solid fuel (P300). Temperature range was 700–850 °C. (**c**) Families of dependencies: voltage U-current density I and power density P vs. current density I, as determined for DC-SOFC (I) with LSM cathode and N_2_ atmosphere over solid fuel (P300). Temperature range was 700–850 °C.

Figure 15a,b present the representative U-I and P-I curves that were recorded for a DC-SOFC that was fueled with ground raw pistachio (P0), the torrefied sample (P300) or charred pistachio shells (P850). The data were recorded for the DC-SOFC (I). Nitrogen was used as a shielding gas in these experimental investigations of DC-SOFC (I).

**Figure 16:**
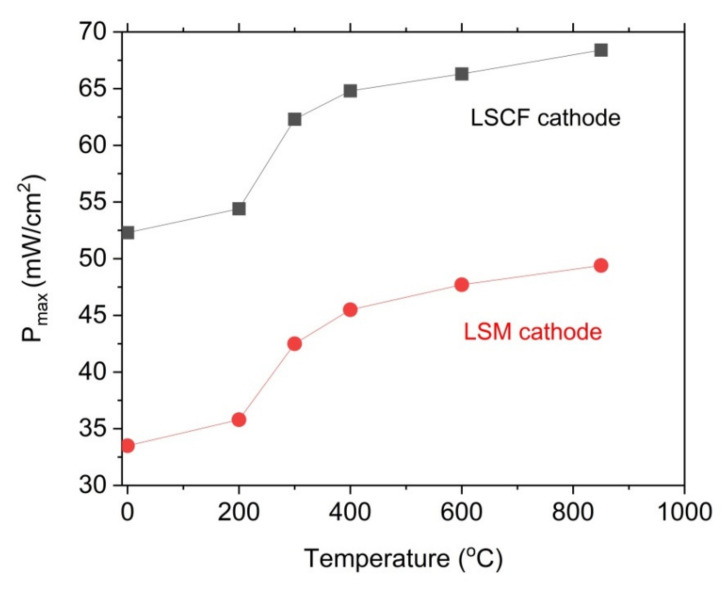
Dependence of maximum P_max_ vs. temperature of solid fuels preparations. P_max_ values were obtained for DC-SOFCs (I) and (II) with LSM or LSCF cathodes, respectively. Data refer to a temperature of 850 °C and experimental conditions that are presented in Figure 15a–c.

As shown in Figure 15a–c, the power output (P_max_) and current density gradually increase with the increase in temperature of the DC-SOFC (I). The effects of the physicochemical properties of the solid fuels that were used with the investigated pistachio shells from P0 to P850 on the performance of the direct carbon fuel cells, varying only the cathode materials used, are shown in Figure 16. The data refer to a temperature of 850 °C

A direct comparison of the results of the power output for the DC-SOFCs (I) and (II) indicated that the higher values of the power output P_max_ were obtained for DC-SOFC (II) with a LSCF cathode compared to DC-SOFC (I), where the LSM cathode was used. These results are directly related to their greater electrochemical activity, which reduces oxygen at the LSCF cathode at lower temperatures than the LSM cathode [63,64]. The kinetics of the oxygen reduction process in direct carbon fuel cells has a significant impact on the performance of the DC-SOFC. These results agree with the observations that were described in a previous article [26].

The main factors that influence the values of the maximum power density P_max_ of DC-SOFCs are the physicochemical properties of the raw ground, torrefied, or charred pistachio shells and the chemical gas composition that formed in the anode chamber. Analyzing the preparation conditions’ influence on the pistachio shells charging the DC-SOFC cells revealed that the lowest values of the power density P_max_ were obtained for solid oxide fuels that were only fueled with ground pistachio shells.

A pyrolysis of the ground pistachio shells in an anaerobic atmosphere led to the formation of torrefied biomass or charred samples. The utilization of such prepared waste-biomass solid fuels in the DC-SOFC led to a gradual improvement of the DC-SOFC’s performance. The increased elemental carbon content and the surface area of the carbon particles are advantageous for the direct electrochemical oxidation of the carbon particles in the anode material Ni-YSZ. Additionally, the indirect gasification of carbon to CO in the anode chamber may affect the electrochemical pathways of anodic oxidation of fuel in the DC-SOFC. A chemical analysis confirmed the presence of CO in the anode chamber in the DC-SOFC and was the only result of the chemical processes, which took place under indirect gasification. This affected the obtained values of the power density P_max_ from the DC-SOFC (I) or (II) cells. These data correspond with the previously observed variations of CO/CO_2_ in a solid carbon bed (Figure 11a) for investigations into carbon solid fuels (P00–P850). Figure 16 shows that the power output P_max_ of the DC-SOFCs (I) and (II) gradually increased alongside the applied thermal treatment of the samples P00 to P850.

The increased thermal treatment of the ground pistachio shells up to 850 °C increased the specific surface area to Sw ~ 377 m^2^/g compared to that of the P600 sample, which obtained the lower value of Sw ~ 180 m^2^/g. The increased carbon content surface area of solid fuels enables a better contact to be obtained between solid carbon grains with an Ni-GDC or Ni-YSZ anode surface and extends the reaction zone of electrochemical oxidation of carbon according to reaction (1) C + 2O_2_^−^→CO_2_ + 4e^−^. Additionally, it improves the performance of the DC-SOFC. These facts indicate that increasing the surface area and carbon content of solid fuels also benefits the performance of the DC-SOFC.

**Figure 17:**
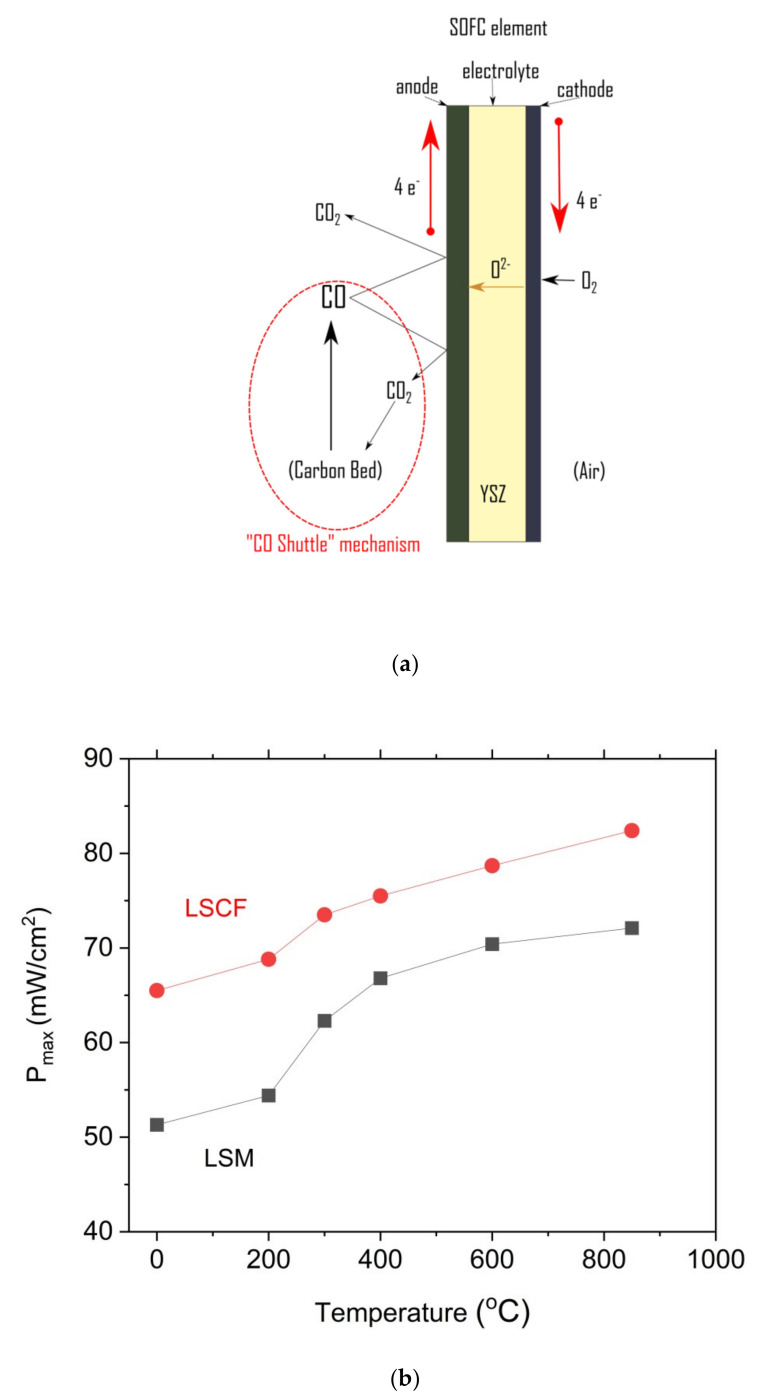
(**a**) Idea of electrochemical oxidation of carbon particles in DS-SOFCs according to CO shuttle mechanism [65]. (**b**) Dependence of P_max_ vs. the temperature of solid fuels preparations. Data were determined for DC-SOFC (I) with an LSM cathode or DC-SOFC (II) with a LSCF cathode and operated at 850°C. CO_2_ gas atmosphere over fuel.

The most common way to explain the performance of the DC-SOFC that is supplied by different carbonaceous-materials is to analyze the electrochemical oxidation mechanism of solid carbons, which is called the “CO shuttle mechanism”. This was previously proposed by Gür [65]. The CO shuttle mechanism (Figure 17a) illustrates the impact of CO, which was first oxidized in the anode of the DC-SOFC to form CO_2_.

The CO_2_, which is a product of the electrochemical oxidation of carbon or CO in the anode, reacts with the carbon bed to produce more CO. Figure 17b suggests that the performance of DC-SOFC can be improved of CO production in solid carbon fuels. There are two main methods of improving the performance of DC-SOFC according to the CO shuttle mechanism idea. The first is adding a catalyst to carbon-based solid fuels, which improves the kinetics of the CO production, according to the Boudouard reaction C + CO_2_→2CO. The second is the introduction of CO_2_ to the anode chamber of the DC-SOFC, where solid carbon fuel is placed. In this way, the generation process is supported.

The effect of the CO_2_ gas atmosphere on solid fuels in the DC-SOFC (I) with LSM or DC-SOFC (II) with LSCF cathodes is presented in Figure 17b.

Introducing CO_2_ as a shielding gas in the DC-SOFC anode chamber leads to much higher power density P_max_ values than using N_2_ as a shielding gas (Figure 17b). One of the reasons for this phenomenon is the possible formation of CO as a product of the Boudouard reaction after the C + CO_2_→2CO reaction. The product of the electrochemical oxidation process is CO_2_ and it has a significant influence on the maximum power density due to the contribution of CO to the electrochemical oxidation of carbon, which proceeds according to the CO shuttle mechanism. The presence of inorganic substances based on alkali oxides and iron promotes the coal gasification reaction in the CO_2_ atmosphere [7,56,57,58]. In our experiments, the addition of CO_2_ to the anode chamber increased the formation of CO and the maximum power density (from approximately 65 mW/cm^2^ to ~83 mW/cm^2^) that was obtained for the DC-SOFC (II) when it was fueled by charred pistachio shells (P850). In this situation, the Boudouard reaction (CO_2_ + C→2CO) that is associated with the electrochemical oxidation of CO on the DC-SOFC results in increased performance. These observations confirmed that the responsible mechanism for the DC-SOFC operation is a shuttle mechanism.

Table 3 data show that the DC-SOFC (I) is supplied by other types of waste-biomass solid fuels.

Table 3 shows that using torrefied or carbonized pistachio shell samples as fuel for the DC-SOFC (I) provides comparable power densities P_max_ in comparison with that of commercially available charcoal and woody biomasses, such as acacia chips. However, they are slightly lower than the power densities when walnut shell-derived samples were used. The results that were obtained for the carbonized samples in hydrothermal conditions were due to the lower temperature that is required when preparing solid fuels [66].

The results of the electrochemical studies on the influence of the physicochemical properties of pistachio shell-based solid fuels on the operating parameters of the DC-SOFC cells do not reflect the full work at this stage, which aims to achieve the highest possible electrochemical parameters of the operation of solid cells. Further directions for research that aims to achieve a P_max_ in the range of 100–400 mW/cm^2^ should focus on selecting catalysts for the electrochemical oxidation of biomass fuels and new groups of anode and cathode materials; optimizing the construction of a single DC-SOFC fuel cell; and expanding the fuel cell stack [67,68,69,70].

The investigation into the impact of humidity on the solid carbon gasification process according to the following chemical reactions (11) and (12) is analyzed:

C + H_2_O = H_2_ + CO (11)

CO + H_2_O = H_2_ + CO_2_ (12)

**Figure 18:**
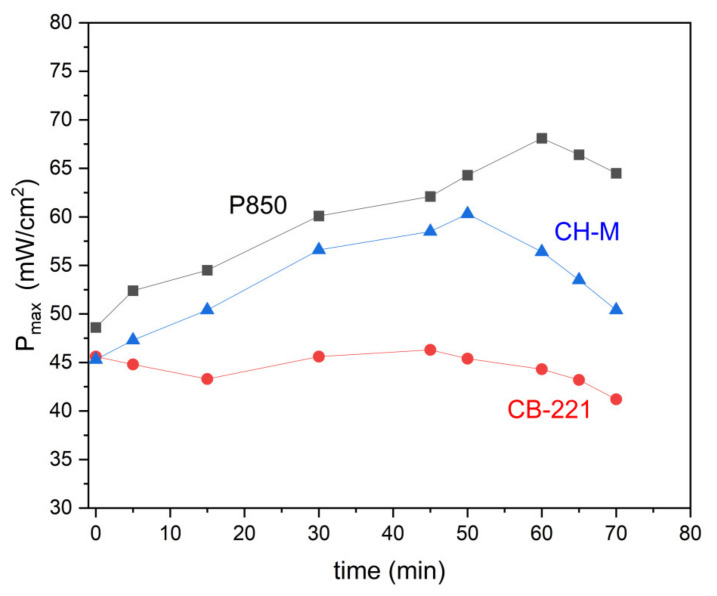
Dependence of P_max_ was recorded for DC-SOFC (I) that was supplied by solid fuel P850 and following solid carbon fuels: carbon black CB-221 and charcoal CH-M. In these experiments, humidified nitrogen was introduced to anode chamber of DC-SOFC (I). Data refer to a temperature of 850 °C.

The gas humidification process was carried out at room temperature by passing a gas stream through the scrubber, which was then directed to the anode space. The U-I and P-I curves were recorded for the DC-SOFC (I) during a period of 70 min. Figure 18 presents the variation of the P_max_ values that were recorded for the DC-SOFC (I), which was fueled with pistachio char P850, a sample of charcoal CH-M or carbon black CB-221, respectively. The data refers to a temperature of 850 °C. The first power output P_max_ of the DC-SOFC (I) was registered immediately following the introduction of humidified nitrogen to the anode chamber of the DC-SOFC (I), and they were further recorded after breaks of less than 10 min, with 15 min for whole duration of the experiment (70 min).

As shown in Figure 18, the P_max_ of the DC-SOFC (I) is observed to gradually increase over a longer period after injecting the humidified nitrogen into it. When applying biochar P850 as a solid fuel to DC-SOFC (I), the gradual increase of power output in P_max_ vs. time may be indicative of the possible gasification of solid fuels over time by steam. The source of the steam is the humified nitrogen, which was introduced to the anode chamber of DC-SOFC (I), where the investigated solid fuel is placed. According to the results presented in Figure 11a, the possible products of gasification for the steamed pistachio P850 sample are H_2_, CH_4_ and CO, which are well-known gaseous fuels for SOFCs. The increased concentration of these gases, especially H_2_ and CO, in the anodic chamber of DC-SOFC (I) is one of the possible reasons for the observed increase in electrical performance of the investigated DC-SOFC (I) supplied by the biochar P850 sample. On the other hand, the observed gradual improvement of power output in Pmax vs. time for DC-SOFC (I), which was supplied by charcoal CH-M, could also correspond to an increase in H_2_ and CO content in the anode chamber of DC-SOFC (I). These results correspond to results presented in Figure 11b. There was no considerable variation of P_max_ vs. time observed in the case of DC-SOFC (I), which utilized Carbon Black 221 as a solid fuel. This type of carbon black sample exhibited minimal reactivity due to H_2_O gasification (Figure 11c).

### 3.3. Postmortem SEM Observation of the Anode Materials after the Electrochemical Test of the DC-SOFC (I)

**Figure 19:**
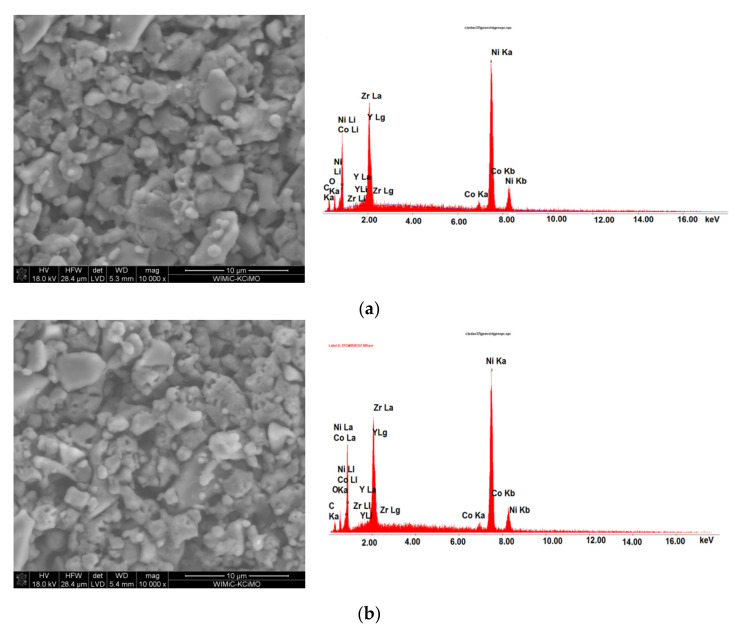
(**a**) SEM image recorded for Ni-GDC|Ni-YSZ anode after electrochemical test with solid fuel P300. (**b**) SEM image recorded for Ni-GDC|Ni-YSZ anode after electrochemical test with solid fuel P850.

A cross-section analysis of the anode-electrolyte interface and surface using SEM observations (Figure 19a,b) took place after the DC-SOFC tests. An EDS analysis was also performed.

No significant changes were observed in either the structure of the surface of the Ni-GDC anode (Figure 19a,b) or its chemical composition compared to that of the starting samples. No areas of corrosive degradation were found on the surface of the Ni-GDC or Ni-GDC|Ni-YSZ|8YSZ anode. These results are in accordance with the X-ray investigations that were performed for the anode materials.

## 4. Conclusions

The paper presents the research results on the characteristics of the physical and chemical properties of pistachio shells as waste biomass in terms of their use as solid fuels. These samples were subjected to the torrefaction and carbonization processes in the temperature range of 200 °C to 850 °C. Based on the tests that were performed, it was found that the thermal treatment process in the temperature range of 400 °C to 850 °C leads to the production of chars with carbon contents of 60% to 90%. In addition to the high carbon content, these fuels have low sulfur and mineral residue content. Other important physicochemical properties of solid fuels include the low degree of graphitization, the isometric shape of the carbon particles, and the sufficient size of the specific surface area of solid fuels. These fuels are characterized by a small share of mineral residue (ash). The main components of the ash, which is obtained by burning writing samples, are periclase, calcite, magnesite, portlandite, and brownmillerite. The analysis of the chemical composition using the EDX and WDXRF methods for powdered samples of solid fuels showed that the share of alkali elements, such as Ca, Mg, K, Sr, and Fe, and inorganic compounds that are contained in the obtained samples, such as solid fuels, are naturally embedded materials catalytic supporting processes of gasification with carbon monoxide (II) or steam vapor. The tests showed that pistachio shells were used as fuel to supply direct DC-SOFCs. The lowest values of P_max_ were reached for the DC-SOFCs (1) or (2), which were supplied by raw pistachio shells. The increased thermal treatment of pistachio shells and their usage in the DC-SOFC provides them with slightly higher current and power densities compared to that of DC-SOFCs that are fueled with torrefied samples. Based on the preliminary research on the gasification of pistachios with carbon dioxide or water vapor, these samples are characterized by significant reactivity towards CO_2_, where the product is carbon monoxide (II). The presence of carbon monoxide (II) as the fuel that is used to power DC-SOFCs is crucial to obtain high current and power densities. The preliminary studies of the steam gasification process of the P850 pistachio charcoal concluded that the steam was characterized by comparable reactivity to commercial charcoal. The observed products of steam gasification are H_2_, CH_4_, and CO. An increased concentration of these gases in the anode chamber of the DC-SOFC for a prolonged period improved their performance.

## Figures and Tables

**Table 1:** Results of elemental analysis (carbon C, hydrogen H, sulfur S, and oxygen O content) for investigated pistachio shells and samples following thermal treatment within a temperature range of 200–850 °C. Determined variations of ash A and moisture M are also included. All data are presented in (mass %).

Sample	C ^d^	H ^d^	(O + N) ^d^	S ^a^	A ^a^	A ^d^	M ^a^
P0	43.1	6.96	49.8	<0.01	0.16	0.18	10.21
P200	48.1	5.38	46.3	<0.01	0.18	0.19	7.32
P300	62.3	3.85	32.9	<0.01	0.85	0.90	5.78
P400	76.2	3.50	19.3	<0.01	0.92	0.96	3.73
P600	87.2	2.23	9.5	<0.01	1.07	1.09	1.82
P850	88.0	1.18	9.5	<0.01	1.36	1.37	0.44

a—data for analytical state; d—data are recalculated to dry state.

**Table 2:** Collected data for analyzed temperature ranges for pyrolysis and gasification stages and weight loss of samples Δm (%) for the samples P400, P600, and P850.

Sample	Pyrolysis		Gasification	
	Temperature Range (°C)	Δm (%)	Temperature Range (°C)	Δm (%)
P400	200–640	27	>640	66
P600	200–670	8	>670	83
P850	200–750	4	>750	71

**Table 3:** Comparison of electrical performance of DC-SOFC (I) for different solid fuels.

Solid Fuel	P_max_ (mW/cm^2^) at 850 °C		Reference
	Intert Gas Atmosphere (N_2_ or Ar)	CO_2_ Gas Atmosphere	
Pistachio shells	~49	~70	This work
Commercial charcoal	~40–65	~60–120	[59]
Charred acacia sample	~55	~65	This work’s data for tubular direct solid oxide fuel cells P_max_ 60–100 mW/cm^2^ [23]
HTC acacia	~62	~75	Electrochemical tests performed under this work. Physical and chemical properties of solid fuels derived from acacia were described in the paper [66]
Walnut shells	~50	~80	[26]
HTC walnut shells	~45	~75	Electrochemical tests performed under this work
Waste coffee grounds	~45	---	[13]
Pretreated mesocarp fibre biochar	~12–18	---	[67]
Carbon Black	~25	~50	[13]

## Data Availability

Not applicable.

## Abbreviations

DC-SOFCs	Direct carbon solid oxide fuel cell technology
XRD	X-ray diffraction analysis method
SEM	Scanning electron microscopy
WDXRF	Wavelength dispersive x-ray fluorescence spectroscopy
DTA	Differential thermal analysis
TG	thermogravimetry
MIR	Mid-infrared spectroscopy
Ni	metallic nickel powder
GDC	5% Gd_2_O_3_ in CeO_2_, solid solutions, gadolina doped-ceria
YSZ	8% Y_2_O_3_ in ZrO_2_ solid solution, cubic zirconia-yttria solid solution
Ni-GDC	cermet anode; 50% vol Ni particles dispersed in 50%vol. GDC oxide matrix
Ni-YSZ	cermet anode; 50% vol Ni particles dispersed in 50%vol. YSZ oxide matrix
P0	raw ground pistachio shells
P200–P850	thermally heated pistachio shell samples; the numbers 200, 300, 400, 600 and 850 represent the temperatures (in Celsius) of the thermal treatment
LSFC	(La_0.60_Sr_0.40_)_0.95_Co_0.20_Fe_0.8_O_3-x_ cathode material
LSM	La_0.8_Sr_0.2_MnO_3_ cathode material
LSFC-GDC	a composite cathode material; 52.4 wt% (La_0.60_Sr_0.40_)_0.95_Co_0.20_Fe_0.80_O_3-x_ and 47.6 wt% Gd_0.10_Ce_0.90_O_1.95_
LSM-GDC	a composite cathode material –53 wt% La_0.8_Sr_0.2_MnO_3_ and 47wt% G_d0.10_Ce_0.90_O_1.95_
